# Domain collapse and active site ablation generate a widespread animal mitochondrial seryl-tRNA synthetase

**DOI:** 10.1093/nar/gkad696

**Published:** 2023-08-28

**Authors:** Bastiaan de Potter, Ingrid Vallee, Noelia Camacho, Luís Filipe Costa Póvoas, Aureliano Bonsembiante, Alba Pons i Pons, Ulrich Eckhard, Francesc-Xavier Gomis-Rüth, Xiang-Lei Yang, Paul Schimmel, Bernhard Kuhle, Lluís Ribas de Pouplana

**Affiliations:** Institute for Research in Biomedicine (IRB Barcelona), The Barcelona Institute of Science and Technology, Barcelona, Catalonia, Spain; Utrecht University Faculty of Science, Department of Biology, Theoretical Biology and Bioinformatics Utrecht, Utrecht, The Netherlands; The Scripps Research Institute, Department of Molecular Medicine La Jolla, CA, USA; Institute for Research in Biomedicine (IRB Barcelona), The Barcelona Institute of Science and Technology, Barcelona, Catalonia, Spain; Institute for Research in Biomedicine (IRB Barcelona), The Barcelona Institute of Science and Technology, Barcelona, Catalonia, Spain; Institute for Research in Biomedicine (IRB Barcelona), The Barcelona Institute of Science and Technology, Barcelona, Catalonia, Spain; Institute for Research in Biomedicine (IRB Barcelona), The Barcelona Institute of Science and Technology, Barcelona, Catalonia, Spain; Molecular Biology Institute of Barcelona, Department of Structural Biology, Barcelona, Catalunya, Spain; Molecular Biology Institute of Barcelona, Department of Structural Biology, Barcelona, Catalunya, Spain; The Scripps Research Institute, Department of Molecular Medicine La Jolla, CA, USA; The Scripps Research Institute, Department of Molecular Medicine La Jolla, CA, USA; The Scripps Research Institute, Department of Molecular Medicine La Jolla, CA, USA; Institute for Research in Biomedicine (IRB Barcelona), The Barcelona Institute of Science and Technology, Barcelona, Catalonia, Spain; ICREA, Catalan Institution for Research and Advanced Studies Barcelona, Catalonia, Spain

## Abstract

Through their aminoacylation reactions, aminoacyl tRNA-synthetases (aaRS) establish the rules of the genetic code throughout all of nature. During their long evolution in eukaryotes, additional domains and splice variants were added to what is commonly a homodimeric or monomeric structure. These changes confer orthogonal functions in cellular activities that have recently been uncovered. An unusual exception to the familiar architecture of aaRSs is the heterodimeric metazoan mitochondrial SerRS. In contrast to domain additions or alternative splicing, here we show that heterodimeric metazoan mitochondrial SerRS arose from its homodimeric ancestor not by domain additions, but rather by collapse of an entire domain (in one subunit) and an active site ablation (in the other). The collapse/ablation retains aminoacylation activity while creating a new surface, which is necessary for its orthogonal function. The results highlight a new paradigm for repurposing a member of the ancient tRNA synthetase family.

## Graphical Abstract

**Graphical Abstract:**
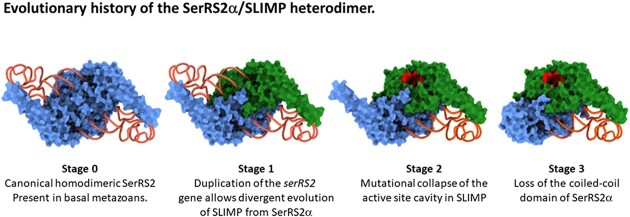

## INTRODUCTION

During gene translation each transfer RNA (tRNA) is aminoacylated with its corresponding amino acid by the cognate aaRS, prior to its transport to the ribosome, where it participates in translation elongation via the recognition of complementary codon triplets in the messenger RNA (1).

The aaRSs are ancient enzymes that evolved contemporarily to the extant genetic code (2), possibly from a primitive two-protein-one-tRNA complex (3). From this process two universally conserved families of approximately ten enzymes each emerged (4). All members of each family share a catalytic domain with a common fold, around which different multi-domain structures evolved. The extended and continuing divergence of aaRSs included the incorporation of additional biological functions that are species-specific and rely on the accretion of new and idiosyncratic domains (5). These non-canonical functions are associated with transcription and translation regulation, nutrient-sensing mechanisms, or eukaryotic regulatory pathways linked to inflammation or cell cycle regulation, among others (6–9).

In eukaryotes, the complex evolutionary history of aaRSs is further confounded by the convergence of two or three orthologous aaRS sets resulting from the endosymbiotic events that gave rise to mitochondria and chloroplasts (10,11). In animals, most cytosolic and mitochondrial aaRSs are encoded by distinct nuclear genes (12). An example of this situation is seryl-tRNA synthetase (SerRS), for which two different nuclear genes exist: *serS*1, coding for the cytosolic enzyme (SerRS1), and *serS2*, which codes for the mitochondria-localized variant (SerRS2) (13).

SerRSs belong to the class II aaRS family (14), and are characterized by the presence of an N-terminal coiled-coil domain that interacts with the pseudo-knot region of the tRNA (15). Homodimeric SerRSs contain two identical tRNA binding sites that span across the dimer inteface, in which the N-terminal coiled-coil domain from one subunit recognizes the tRNA pseudoknot, while the tRNA acceptor stem binds to the catalytic site of the second subunit. Thus, each monomer contributes its coiled-coil domain to one tRNA binding site, and its catalytic site to the opposite tRNA binding site (15–17).

The maintenance of mitochondria-specific SerRS subsets is required to aminoacylate mitochondrial tRNA^Ser^ (mt-tRNA^Ser^) which are exposed to distinct selective pressures and elevated evolutionary rates, leading to a marked divergence characterized by the loss of structural features that are usually highly conserved in canonical cytosolic tRNAs (18–20). The mt-tRNA^Ser^_GCU_ represents one of the most prominent examples for this process, undergoing loss of the long variable (V)-arm, deletion of the entire D-arm, and a substantial remodelling of its T-arm. Biochemical and structural work shows that the degeneration of tRNA^Ser^_GCU_ was accompanied by a fundamental rewiring of the intermolecular specificity rules underlying SerRS/tRNA^Ser^ interactions in mammals (17,21,22), possibly explaining why dedicated SerRS2 are retained.

**Figure 1.**
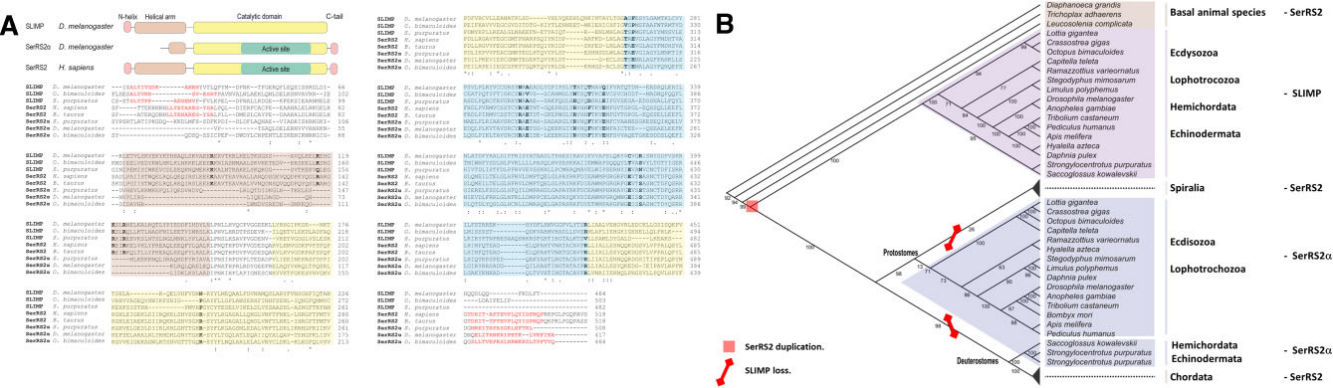
(**A**) Multiple sequence alignment of sequences of SerRS2, SerRS2α, and SLIMP. Residue conservation is marked by dots and asterisks. Functionally relevant positions are shown in bold. The regions that correspond to the major domains of each protein type are represented by colored blocks under the alignment, and the sequences are color-shadowed accordingly. (**B**) Phylogenetic analysis of SerRS2, SerRS2α and SLIMP sequences performed as described. Numbers at branches represent the bootstrap support for each node. The deduced point of SerRS2 duplication that gave rise to SerRS2α and SLIMP is marked by a red square. The branches where SLIMP was lost are crossed by red lines to signify the gene loss (see also Supplementary Figure S1).

In animals, a second paralogous mitochondrial *serS* gene that codes for the protein SLIMP (Seryl-tRNA synthetase-Like Insect Mitochondrial Protein) evolved following the duplication of a *serS2* gene early in the evolution of metazoans, and was retained by most animal groups but lost in vertebrates (23). For clarity, here we will use SerRS2 to refer to all mitochondrial SerRS. In SLIMP-containing species we will refer to the subunits SerRS2α and SLIMP, to illustrate the fact that these two proteins may form a heterodimer (SLIMP/SerRS2α) (Figure 1A).

In *Drosophila melanogaster*, SLIMP (*Dm*SLIMP) is an essential protein that associates with SerRS2α (*Dm*SerRS2α) forming an obligate heterodimer (*Dm*SLIMP/*DmSerRS2α*) absolutely required to aminoacylate mt-tRNA^Ser^. *Dm*SLIMP depletion induces a loss in respiratory capacity and a distorted mitochondrial morphology in all tissues and developmental stages (23–25). Remarkably, the *Dm*SLIMP/*Dm*SerRS2α complex is involved in complex regulatory pathways that control mitochondrial DNA replication via an interaction with the LON protease (25), and cell cycle progression from G1 to S phase (26,27).

In order to understand the evolution of metazoan SerRS2, the structural differences between homodimeric SerRS2 and the SLIMP/SerRS2α heterodimer, and the ability of *Dm*SLIMP/*Dm*SerRS2α to retain full recognition of tRNA^Ser^ while gaining new cellular functions, we characterized the phylogenetics and structural evolution of these proteins, and experimentally studied their tRNA recognition mechanisms. These analyses unveiled a remarkable assymetric co-evolution of SLIMP and SerRS2α in all SLIMP-containing species, indicating that the heterodimer described in *Drosophila* is likely the enzyme variant active in most invertebrates. Most striking in this co-evolution of the two subunits is the concomitant loss of the N-terminal coiled-coil domain in the SerRS2α subunit and the collapse of the catalytic site of SLIMP.

We show here that the coiled-coil domain in *Dm*SLIMP remains indispensable for aminoacylation, demonstrating that the *Dm*SLIMP/*Dm*SerRS2α complex has a single aminoacylation site. Notably, the mechanism of tRNA^Ser^_GCU_ recognition by *Dm*SerRS2 is very similar to that of homodimeric mammalian SerRS2s, even allowing cross-species aminoacylation of human mt-tRNA^Ser^_GCU_ by the *Drosophila* enzyme. Finally, we show that the aminoacylation activity of the *Dm*SLIMP/*Dm*SerRS2α heterodimer is compatible with the binding of additional molecular partners.

## MATERIALS AND METHODS

### Phylogenetic analyses

For phylogenetic inferences we initially retrieved the sequences of SerRS2 homologs from the National Center for Biotechnology Information (NCBI) (28), or identified them by BLAST searches. Subsequently, homologs were aligned with Clustal_X (Supplementary Figure S2) (29). Phylogenetic analyses were performed using distance methods and maximum likelihood (PROTDIST and PROTMLg) with PHYLIP 3.6 (30). The program SEQBOOT was used to create 100 bootstrap replicates of the initial alignments. Subsequently, NEIGHBOR and CONSENSE were used to calculate the confidence limits of each node using the 100 bootstrap replicates. Trees were drawn with DRAWGRAM and DRAWTREE, and visualized with Adobe Illustrator.

We then additionally performed a phylogenetic pipeline on a local database (Supporting data, excel file) (31). Eukaryotic homologs were identified using pairwise searches with BLASTP v.2.12.0+ (32) against our local proteome database (Supporting data, excel file) with full length *Homo sapiens* SerRS2 (*Hs*SerRS2) as a query (Uniprot: Q9NP81). Significant hits (threshold E < 10–5) were aligned using MAFFT v.7.490 (settings: genafpair, maxiterate = 1000) and processed with trimalAl v1.4.rev15 [gt = 0.1]) (33,34). Phylogenetic trees were inferred with IQ-TREE v.2.1.4 (ModelFinder, ultrafast bootstrap = 1000) (35), and subsequently visualized and annotated using iTOL (Supplementary Figure S1) (36).

### Structure modeling and analysis

3D protein structures for both SLIMP and SerRS2 sequences were created by means of ExPASy SWISS-MODEL homology modeling, with the crystallized *Bos taurus* SerRS2 (*Bt*SerRS2) structure as a template for all sequences (pdb: 1WLE) (23,30). As of recently however, these structures can also be found in the AlphaFold EBI database v2. To create the heterodimer, Alphafold2 (37) was operated through ColabFold (38). 3D structures were accordingly analysed by comparing the structural features, the globular domain and coiled-coil, in the structural alignments with BtSerRS2. A coiled-coil determination was performed for all the sequences with MARCOIL (39,40).

### Active site analysis

An analysis of the residues potentially involved in the interaction with seryl-adenylate in the active site of SLIMP and SerRS2 was based on multiple sequence alignments as described by Guitart et al. (26). Sequences were aligned with Clustal_X.

### Determination of conserved residues involved in tRNA^Ser^ recognition

Models complexed with tRNA were created with PYMOL for both a *Bt*SerRS2 homodimer and for a *Dm*SLIMP*/DmSerRS2α* heterodimer. Structural differences between the canonical SerRS2 homodimer and SLIMP/SerRS2α heterodimer were identified by aligning the structures. Amino acid residues potentially involved in tRNA recognition and binding were identified according to the tRNA configuration relative to the models. To analyse amino acid conservation, a multiple sequence alignment was performed for SerRS2 and SLIMP sequences with Clustal_X. tRNA^Ser^ structures were compared and structural alignments were created to identify differences in tRNA^Ser^ between species containing the canonical SerRS2 homodimer and species containing SLIMP.

### Protein cloning and purification

*Dm*SLIMP, *Dm*SerRS2α and Δ*Dm*SLIMP were cloned into the pQE-70 vector (QIAGEN) for bacterial expression of a C-terminal 6His-tagged protein. Δ*Dm*SLIMP was found to be mostly insoluble, which significantly reduced the purification yield of this protein (Supplementary Figure S3). The sequence of all plasmids was confirmed by sequencing. *Dm*SLIMP and *Dm*SerRS2α were cloned into pOPINFS expression vectors (Oxford Protein Production Facility) as a bicistronic product, resulting in a C-terminal 6His-tagged *Dm*SerRS2α and C-terminal Strep II tagged *Dm*SLIMP separated by a ribosome binding site. BL21 (DE3) cells (Nzythech) were transformed with the resulting plasmids and a starter culture was diluted 1/100 in autoinduction media at 37°C for 2 hours, and 17 hours at 25°C until the OD600 was stable. After harvest, the cell pellet was resuspended in lysis buffer (20 mM sodium phosphate buffer, 200 mM NaCl, 50 mM imidazole, protease inhibitor cocktail, and DnaseI) and lysed using a cell disruptor (20 000 Psi). The lysate was centrifuged at 24 000 *g* for 1 h, and the supernatant was filtered with a 0.45 mm filter. The enzymes were purified on HisTrap columns according to the manufacturer's protocol (GE Healthcare Life Sciences). The collected fractions were analyzed by SDS-PAGE and dialyzed (20 mM sodium phosphate buffer, 200 mM NaCl, 1 mM DTT). Proteins were stored in dialysis buffer with 10% glycerol, and protein concentration was measured with Pierce BCA Protein Assay Kit (Thermo Fisher).

### Expression and purification of the mitochondrial lon protease substrate binding domain (lon-SBD)

The substrate binding domain of the mitochondrial Lon protease isoform from *Drosophila melanogaster* (Lon-SBD; UniProt ID Q7KUT2-2; His93 to Ile432) was cloned into the pCri4a protein expression plasmid (Addgene plasmid #61314) coding for an N-terminal 6xHis-tagged thioredoxin domain, which can be removed upon incubation with Tobacco Etch Virus (TEV) protease.

Protein was expressed in *Escherichia coli* strain BL21(DE3), and protein production induced at an OD_600_ of 1.2 to 1.5 using 0.5 mM isopropyl β-d-1-thiogalactopyranoside (IPTG; Fisher Bioreagents). Cultures were subsequently transferred to 20°C for overnight expression (∼20 h), and harvested by centrifugation (4000 RCF, 20 min, 4°C). Typical cell pellets were 4–5 g/l of culture, which were resuspended in ∼5 ml of cold Buffer A (50 mM Tris–HCl, 150 mM NaCl, pH 8.0) supplemented with ‘Complete’ Protease Inhibitor Cocktail (without EDTA; Roche Life Sciences) per gram of pellet. Cells were lysed by one round of sonication, adding 10 μg/ml Dnase I (Roche Life Sciences), and one cell disrupter (Constant Systems) passage at 135 mPa. Soluble protein was clarified by centrifugation (25 000 RCF, 20 min, 4°C), and subsequently passed through a 0.22-μm filter (Merck Millipore).

For immobilized-metal affinity chromatography (IMAC), clarified lysates were supplemented with 10 mM imidazole, and loaded onto a pre-equilibrated gravity flow column containing 1.5 ml of Ni Sepharose 6 Fast Flow resin (Cytiva), washed and eluted with Buffer A containing 20 and 250 mM imidazole, respectively. Elution samples were then rebuffered to Buffer A using a Vivaspin 20 Ultrafiltration Unit (MWCO 10 0000, PES. Sartorius) to remove imidazole. Samples were then incubated with in-house produced 6His-tagged TEV protease at a peptidase to substrate ratio of 1:50 (w/w) in presence of 5 mM β-mercaptoethanol at 4°C overnight. The sample was then reloaded onto the same column equilibrated in Buffer A to remove uncleaved target protein and TEV protease. The flow-through of the reverse chromatography was re-concentrated and further purified using size exclusion chromatography and a Superdex 200 GL column (Cytivia). Protein purity was verified by SDS-PAGE analysis.

### In vitro transcription of tRNAs

tRNAs were obtained by *in vitro* transcription using T7 RNA polymerase according to standard protocols (Saint-Léger et al., 2016). Genes encoding tRNA^Ser^ variants were cloned into the pUC-19 vector under the control of a T7 RNA polymerase promoter. DNA-templates for *in vitro* transcription were amplified by PCR using forward and reverse primers complimentary to the T7 promoter and the 3′ end of the tRNA gene, respectively. Transcription reactions were performed in 40 mM Tris–HCl pH 8.0, 25 mM NaCl, 25 mM MgCl_2_, 2 μg/ml yeast pyrophosphatase (Roche), 1 mM Spermidine, 5 mM DTT, 18 mM GMP, 4 mM each of ATP, CTP, GTP and UTP with 75 μg/ml T7 polymerase and DNA template at 37°C for 6 hours. Reactions were stopped by phenol/chloroform extraction followed by purification of the tRNA by 12% denaturing PAGE. tRNA was eluted from the gel in buffer containing 200 mM NaOAc, 20 mM Tris–HCl, 5 mM EDTA (pH 5.3), followed by ethanol-precipitation. The final tRNA was taken up in Rnase-free water and stored at –80°C.

### Aminoacylation assays

*In vitro* tRNA aminoacylation assays were performed at 25°C in 100 mM HEPES pH 7.2, 40 mM KCl, 50 mM MgCl_2_, 1 mM ATP, 0.1 mg/ml^–1^ BSA, 1 mM DTT, 10 mM serine, 500 Ci/mol – l–[^3^H(G)]-Serine and 0.5 mM protein. Reactions were initiated by addition of pure enzyme and samples of 22 μl were spotted onto Whatman 3 Mm discs at varying time intervals. Radioactivity (corresponding to amino acid ligated to tRNA substrate) was measured by liquid scintillation (TriCard 2900TR, Packard).Aminoacylation reactions with mutant SerRS and tRNAs were carried out in an assay solution containing 50 mM HEPES pH 7.5, 60 mM KCl, 20 mM MgCl_2_, 4 mM ATP, 2 mM DTT, 4 μg/ml pyrophosphatase, 10 μM cold l-serine and 5 μM [^3^H]-serine (1 mCi/ml). Reactions were initiated by addition of SerRS (0.5 μM). At varying time intervals, 5-μl aliquots were removed and applied to a MultiScreen 96-well filter plate (0.45 μm pore size hydrophobic, low-protein-binding membrane; Merck Millipore), pre-wetted with quench solution (0.5 mg/ml salmon sperm DNA, 0.1 M EDTA, 0.3 M NaOAc (pH 3.0)). After all time points were collected, 100 μl of 20% (w/v) trichloroacetic acid (TCA) was added to precipitate nucleic acids. The plate was washed four times with 200 μl of 5% TCA containing 100 mM cold serine, followed once by 200 μl of 95% ethanol. The plate was then dried, followed by deacylation of bound tRNAs by addition of 70 μl of 100 mM NaOH. After 10 min incubation at RT, the NaOH-solution was centrifuged into a 96-well flexible PET microplate (PerkinElmer) with 150 μl of Supermix scintillation mixture (PerkinElmer). After mixing, the radioactivity in each well of the plate was measured in a 1450 MicroBeta Micoplate Scintillation and Luminescence Counter (PerkinElmer).

## RESULTS

### Phylogenetics of SerRS2, SerRS2α and SLIMP

To study the evolution of metazoan SerRS2 we performed full length alignments of animal SerRS sequences and performed phylogenetic analyses (31) which confirmed the existence of two major clades: one populated by cytosolic SerRS, and the second containing mitochondrial SerRS and SLIMP sequences (Figures 1a–b, and Supplementary Figure S1). The SLIMP sequences are monophyletic and emerge early in metazoan evolution, after a duplication that took place at the root of bilaterian species. Interestingly, SLIMP evolves faster than SerRS2, but is retained in most invertebrate taxa, including arthropods, molluscs, annelids, tardigrades, echinoderms, and hemichordates (Figures 1A, B, and Supplementary Figure S1). This phylogenetic analysis reveals the distribution of potential SLIMP/SerRS2α heterodimers in metazoans.

### The SerRS catalytic site pocket is lost in SLIMP sequences

**Figure 2.**
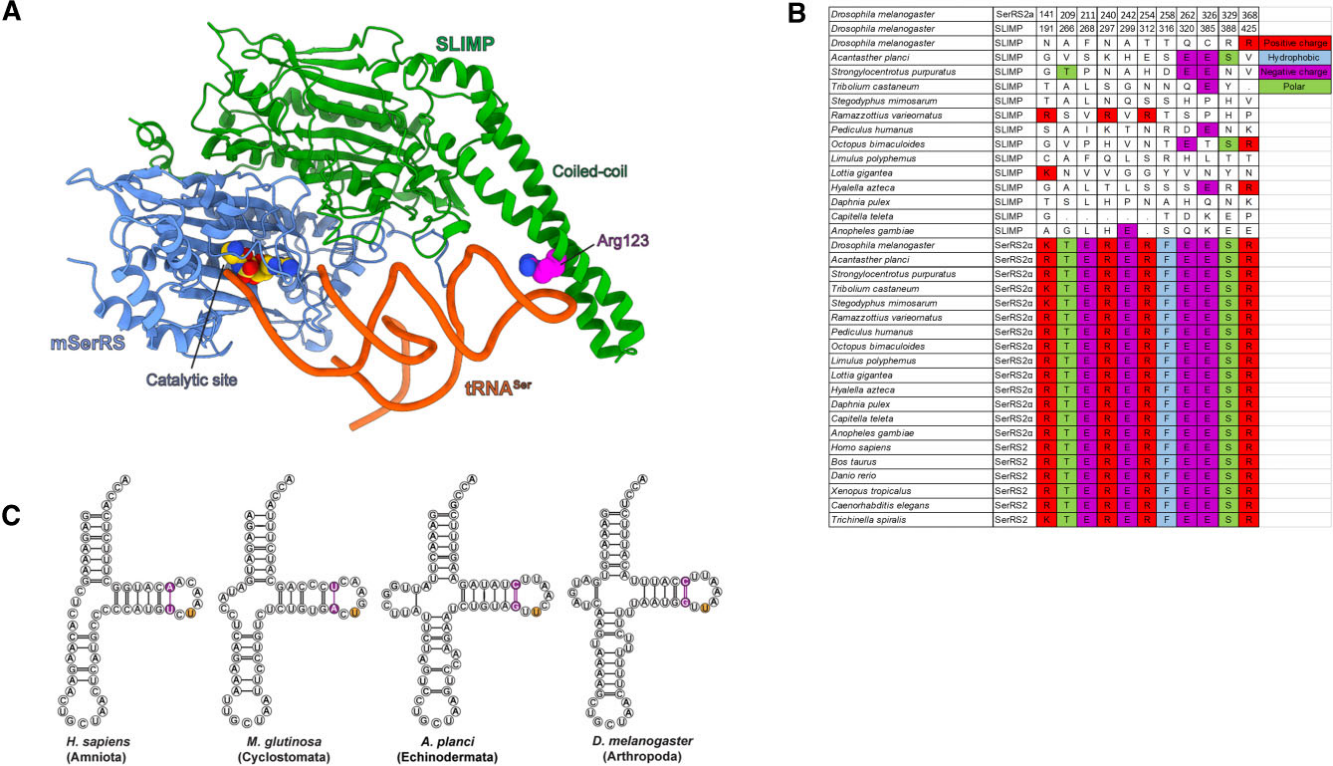
(**A**) Three-dimensional homology-based model of the *Dm*SLIMP/*Dm*SerRS2α heterodimer bound to a tRNA molecule. The *Dm*SLIMP subunit is shown in green, *Dm*SerRS2α in blue and the tRNA in red. Residues in the catalytic pocket of SerRS2α, and the conserved arginine in the coiled coil domain of *Dm*SLIMP are displayed in CPK format. Notice the absence of a coiled-coil domain in *Dm*SerRS2α, and the presence of a single tRNA molecule bound by the two enzyme subunits. (**B**) Alignment of the sequences of the catalytic pockets of SerRS2, SerRS2α and SLIMP sequences. (**C**) Secondary structure representation of animal tRNA^Ser^_GCU_ structures illustrating the overall conservation of these molecules.

Whole-sequence structure-based alignments of *Dm*SLIMP and *Dm*SerRS2α with *Bos taurus* SerRS2 (80.5% and 89.6% sequence identity, respectively) show that the overall structure of the catalytic core domain of class II aaRSs is well conserved in both *Dm*SLIMP and *Dm*SerRS2α (Supplementary Figure S2). To study the detailed structural features of the *Dm*SLIMP/*Dm*SerRS2α heterodimer we used the structure-based alignments to model its 3D structure using Alphafold Multimer (41) (Figure 2A), and manually docked a tRNA molecule using the complex structure of *H. sapiens* SerRS2 with mt-tRNA^Ser^_GCU_ (PDB: 7U2B) as a reference (17).

This analysis shows that, while all SerRS2α conserve the canonical catalytic site architecture, SLIMP sequences present a complete collapse of the catalytic cavity due to the loss of all conserved residues in the catalytic pocket and adjacent positions (Figure 2B). This degeneration includes mutations at positions involved in the binding of the seryl-adenylate, such as N297 and T316 (R240 and F258 in DmSerRS2α, respectively), or the acceptor stem of tRNA^Ser^, such as N191 and T312 (K141 and R254 in DmSerRS2α, respectively) (Figure 2B). These observations are in agreement with the fact that *Dm*SLIMP on its own is unable to bind ATP or aminoacylate tRNA^Ser^ (23). This catalytic site degeneration occurs in all SLIMP sequences, suggesting an extended distribution of the SLIMP/SerRS2α heterodimer among invertebrates. Interestingly, in the starfish *Acanthaster planci* conservation of catalytic site residues in SLIMP is slightly higher, a first indication that evolution of SLIMP/SerRS2α in echinoderms may differ from other species (see below) (Figure 2B).

These observations, together with the fact that *Dm*SLIMP is required for aminoacylation in *Drosophila*, suggest that SLIMP/SerRS2α heterodimers present a single tRNA binding surface, where aminoacylation takes place at the catalytic site of the SerRS2α subunit. To try to identify whether tRNA^Ser^ present idiosyncratic features adapted to the SLIMP/SerRS2α complex, we built structure-based alignments of animal tRNA^Ser^ sequences. However, with the exception of a small insertion in the D-arm of tRNA^Ser^_GCU_, we found mt-tRNA^Ser^ in SLIMP-containing organisms to be generally similar to other mt-tRNA^Ser^ (Figure 2C and Supplementary Figure S3).

### The coiled-coil domain is lost in SerRS2α but retained in SLIMP

In vertebrate SerRS2, tRNA recognition is achieved primarily through interactions with three distinct structural domains, namely: the ‘coiled-coil’, the ‘N-helix’, and the ‘C-tail’ domains (22) (17). The coiled-coil domain is the major tRNA^Ser^ recognition element in SerRSs and contacts the identity-defining extended V-arm of cytosolic tRNA^Ser^ as well as the D- and T-loops of mammalian mt-tRNA^Ser^ variants (22,42) to properly position the tRNA molecule relative to the catalytic site. The N-helix and C-tail represent mitochondria-specific sequence extensions, to date only described in vertebrate SerRS2s, where they form an interface with the T-stem to promote tRNA binding and aminoacylation (22,17).

Our model of the *Dm*SLIMP/*Dm*SerRS2α heterodimer shows that *Dm*SLIMP conserves the N-terminal coiled-coil domain characteristic of canonical SerRS structures. Strikingly, however, this coiled-coil domain is completely lost in *Dm*SerRS2α (Figures 2A, B, and Supplementary Figure S2). Thus, the degeneration of the catalytic site pocket in *Dm*SLIMP is accompanied by the loss of the coiled-coil domain in *Dm*SerRS2α. This asymmetric domain loss extends to the N-terminal extension (lost in *Dm*SerRS2α and retained by *Dm*SLIMP), and to the C-tail domain (lost in *Dm*SLIMP and retained by *Dm*SerRS2α) (Supplementary Figure S2).

**Figure 3.**
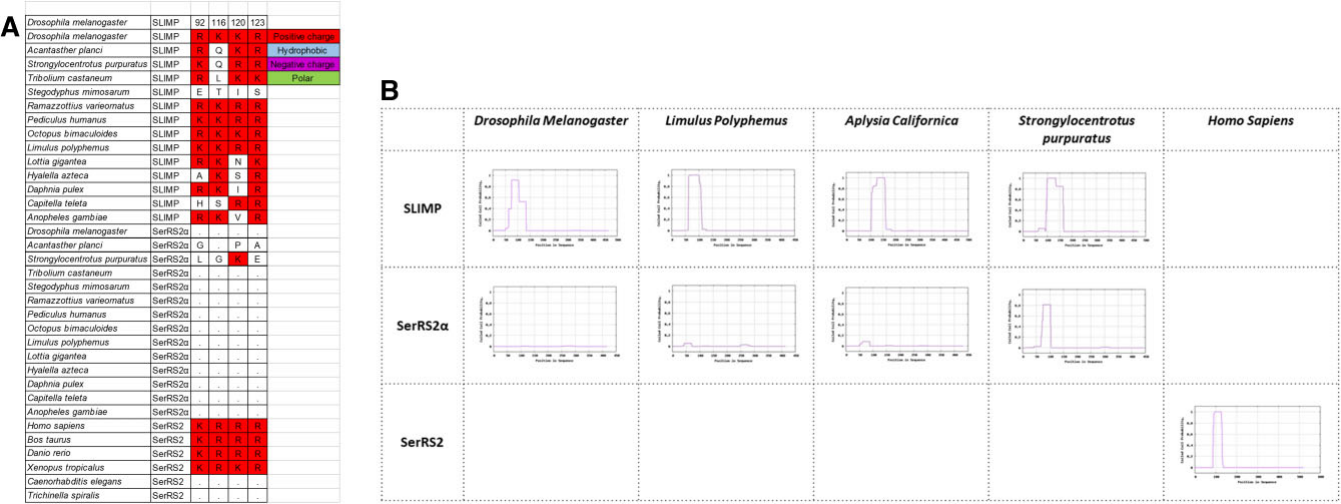
(**A**) Sequence alignment of most conserved positions in the coiled-coil domains of SerRS2, SerRS2α and SLIMP sequences, showing the disappearance of these positions in SerRS2α. The corresponding sequence numbering for *Dm*SerRS2α and *Dm*SLIMP are shown in the top two rows (see also Supplementary Figure S2). (**B**) Predictions of coiled-coil structures in representative sequences of SerRS2, SerRS2α and SLIMP.

We used the coiled-coil prediction algorithm MARCOIL (39,40) to determine the presence or absence of the coiled-coil structure in SLIMP and SerRS2α across species (Figure 3B). This revealed that the concomitant loss of the coiled-coil domain in SerRS2α and the collapse of the catalytic site pocket in SLIMP extends to all SLIMP-containing species. Interestingly, the echinoderm *Strongylocentrotus purpuratus* SerRS2α retains a vestigial coiled-coil structure. This, together with the conservation of some SLIMP catalytic site residues in the starfish *A. planci* (*vide supra*), suggests that *DmSLIMP*/*Dm*SerRS2α evolution in echinoderms differs from other invertebrates.

### SLIMP’s coiled-coil domain is required for tRNA recognition and aminoacylation

The coiled-coil of SerRS is essential for tRNA^Ser^ recognition, and the critical residues involved in this function have previously been identified for *B. taurus* and *H. sapiens* SerRS2 (22,17). Our data show that these positions are conserved throughout most SLIMP sequences, while they are lost along with the coiled-coil domain in most SerRS2α (Figure 3A). Moreover, we have previously shown that purified *Dm*SLIMP or *Dm*SerRS2α alone do not possess aminoacylation activity, while their co-expression generates a heterodimeric *Dm*SLIMP/*Dm*SerRS2α capable of efficiently charging mt-tRNA^Ser^ with serine. Similarly, reconstituting an enzymatically active heterodimeric structure from individually purified *Dm*SLIMP and *Dm*SerRS2α is possible (25).

**Figure 4.**
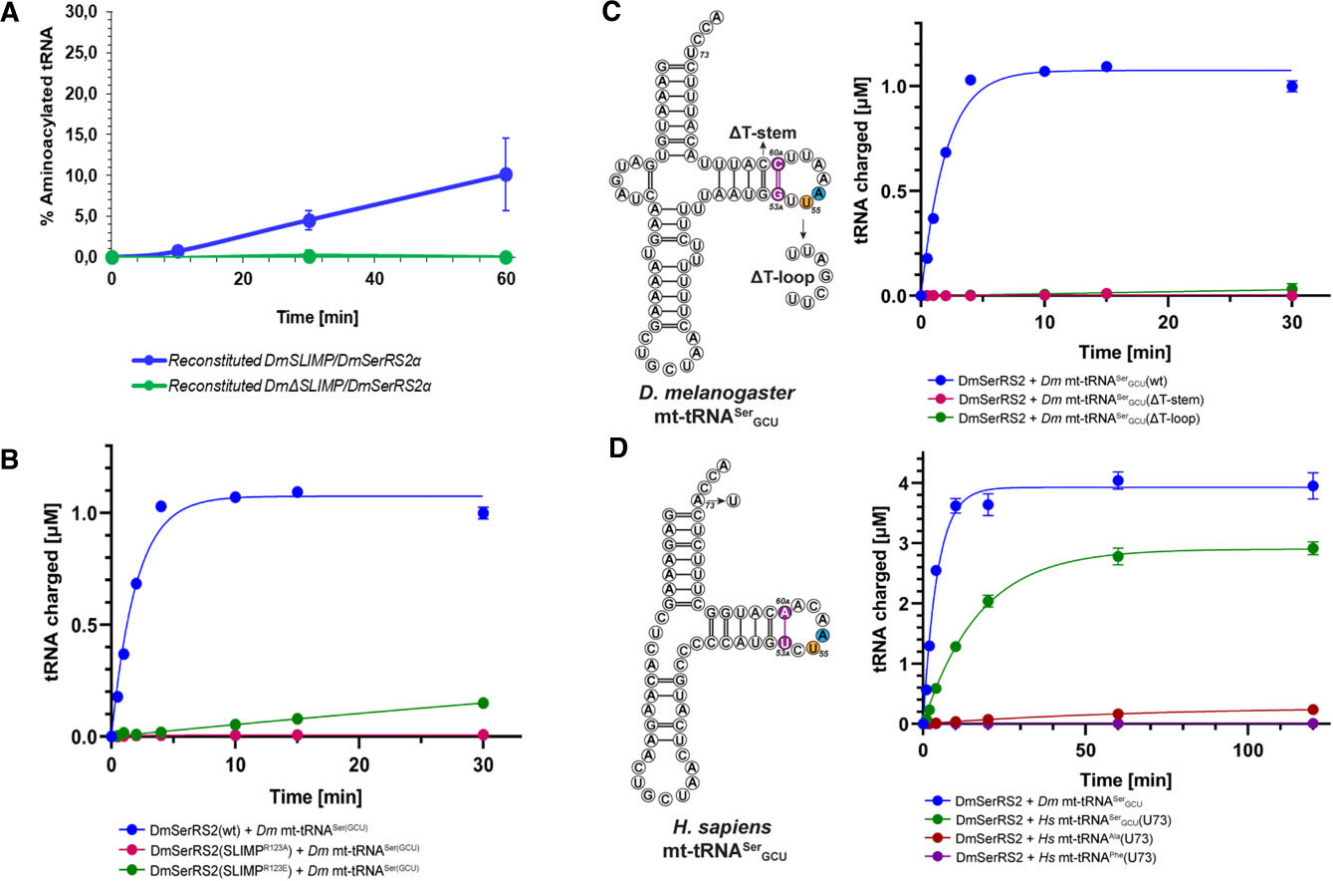
(**A**) Aminoacylation assays of mt-tRNASerGCU by reconstituted DmSLIMP/DmSerRS2α and DmSerRS2α/DmDSLIMP heterodimers (see also Supplementary Figure S3). (**B**) Aminoacylation assays of mt-RNASerGCU by DmSLIMP/DmSerRS2α, DmSLIMP/DmSerRS2αR123A, and DmSLIMP/DmSerRS2αR123E. (**C**) Aminoacylation of mt-tRNASerGCU variants by DmSLIMP/DmSerRS2α in which either the T-stem was reduced from 6 base-pairs to the canonical 5 bp (Dm tRNASer(GCU)(DT-stem)), or the non-canonical T-loop was replaced by a canonical one (Dm tRNASer(GCU)(DT-loop)). (**D**) Aminoacylation of human U73-tRNAs by DmSLIMP/DmSerRS2α.

To experimentally evaluate the functional importance of the coiled-coil domain of SLIMP, we generated a variant of *Dm*SLIMP that lacks the N-terminal coiled-coil domain (Δ*Dm*SLIMP, see materials and methods), and tested its ability to support tRNA^Ser^ aminoacylation *in vitro*. The Δ*Dm*SLIMP/*Dm*SerRS2α heterodimer was not efficiently expressed and could not be purified. We then attempted to reconstitute the Δ*Dm*SLIMP/*Dm*SerRS2α heterodimer from individually purified *Dm*SerRS2α and Δ*Dm*SLIMP (Supplementary Figure S3). In contrast to reconstituted *Dm*SLIMP/*Dm*SerRS2α, the reconstituted Δ*Dm*SLIMP/*Dm*SerRS2α heterodimer showed no aminoacylation activity of *Drosophila* mt-tRNA^Ser^ (Figure 4A).

Biochemical and structural analyses have shown that mammalian SerRS2s use a conserved arginine (R146 in human SerRS2) in the coiled-coil domain to specifically recognize the idiosyncratic T-arm architecture of mt-tRNA^Ser^_GCU_. Notably, sequence comparisons show that this arginine is conserved in SLIMP (R123). We generated R123 mutants of *Dm*SLIMP and analysed their effect on cognate tRNA^Ser^_GCU_ aminoacylation. Both *Dm*SLIMP^R123A^ and *Dm*SLIMP^R123E^ abolished aminoacylation by the *Dm*SLIMP/*Dm*SerRS2α heterodimer, the same effect described for the corresponding R146 mutations in bovine and human SerRS2 (22) (17) (Figure 4B). Thus, the *Dm*SLIMP coiled-coil domain is essential for tRNA aminoacylation, possibly through a mechanism that is conserved between homodimeric and heterodimeric SerRS2.

### The tRNA recognition by *Dm*SLIMP/*Dm*SerRS2α is largely conserved with human SerRS2

As shown above (Figure 2C), a comparison of metazoan tRNA^Ser^_GCU_ sequences shows no apparent major difference between SLIMP-containing species and those devoid of SLIMP. For example, the T-arm architecture of human mt-tRNA^Ser^_GCU_ (a key structural feature that includes a (6-bp) T-stem and a remodelled non-canonical T-loop) (17) is conserved in *Drosophila* tRNA^Ser^_GCU_ (Figure 2C). This led us to hypothesize that the tRNA recognition mechanism may be conserved between hetero- and homodimeric SerRS2.

We tested the ability of heterodimeric *DmSLIMP/DmSerRS2*α to aminoacylate mt-tRNA^Ser^_GCU_ variants in which either the T-stem was reduced from 6 base-pairs to the canonical 5 bp (*Dm* tRNA^Ser(GCU)^(DT-stem)) or the non-canonical T-loop was replaced by a canonical one (*Dm* tRNA^Ser(GCU)^(DT-loop)). Both mutations abolished charging of the tRNA^Ser^_GCU_ variants, demonstrating that the non-canonical structural features of mt-tRNA^Ser^_GCU_ recognized by mammalian SerRS2 are also important for recognition by heterodimeric *Dm*SLIMP/*Dm*SerRS2α (Figure 4C) (17).

Then we asked whether heterodimeric *Dm*SLIMP/*Dm*SerRS2α could cross-aminoacylate human mt-tRNA^Ser^. However, virtually no charging activity was observed using a wild-type transcript of human mt-tRNA^Ser^_GCU_ (data not shown). Mammalian SerRS2 recognizes the discriminator base (N73) of mt-tRNA^Ser^ with a preference for purine bases (21,43), whereas most invertebrate mt-tRNA^Ser^ sequences contain a U73. We generated a human mt-tRNA^Ser^_GCU_(A73U) mutant. Strikingly, this transcript was efficiently charged by *Dm*SLIMP/*Dm*SerRS2α (Figure 4D).

We also tested whether heterodimeric *Dm*SLIMP/*Dm*SerRS2α could aminoacylate human mt-tRNA^Ala^(A73U) or mt-tRNA^Phe^(A73U) transcripts, but no activity could be detected with these tRNAs, demonstrating that the heterodimeric *Dm*SLIMP/*Dm*SerRS2α can efficiently discriminate against non-cognate human mt-tRNAs (Figure 4D).

Taken together, these results support the hypothesis that the *Dm*SerRS2α subunit of *Dm*SerRS2 recognizes the discriminator base of cognate tRNA^Ser^ with a preference for pyrimidine bases, and that the coiled-coil domain of *Dm*SLIMP specifically binds the idiosyncratic T-arm of mt-tRNA^Ser^_GCU_ using the same recognition mechanism as homodimeric mammalian SerRS2 (Figures 4B–D).

### *Dm*SerRS2α/*Dm*SLIMP aminoacylation activity is compatible with LON binding

The evolutionary loss of the coiled-coil domain in *Dm*SerRS2α and the degeneration of the catalytic site pocket of *Dm*SLIMP necessarily generates a heterodimeric enzyme in which only one tRNA binding surface is used, thus liberating surface area for interactions with other proteins.

**Figure 5.**
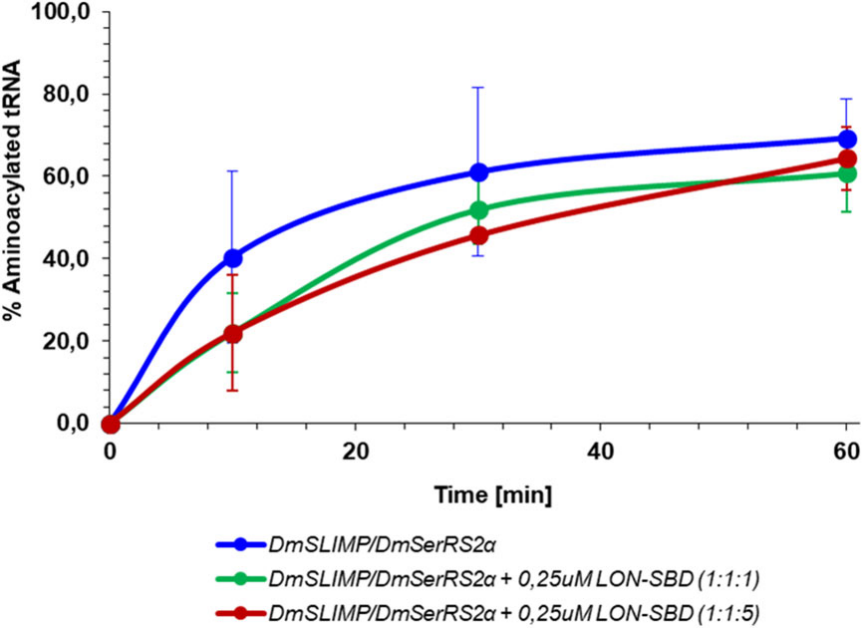
Aminoacylation assays of mt-tRNA^Ser^_GCU_ by *Dm*SLIMP/*Dm*SerRS2α in the presence or absence of LON’s substrate binding domain at two different relative concentrations.

Previous reports demonstrated that SLIMP interacts with the substrate binding domain of the mitochondrial protease LON to stimulate the degradation of TFAM (25). To test whether the interaction of LON with heterodimeric *Dm*SLIMP/*Dm*SerRS2α is compatible with the aminoacylation activity of the enzyme, we performed aminoacylation assays with co-expressed and reconstituted *DmSLIMP*/*Dm*SerRS2α (*vide supra*) in the absence or presence of LON’s substrate binding domain. In all cases the aminoacylation activity of *Dm*SLIMP/*Dm*SerRS2α was not affected by the presence of the LON domain in the reaction mixture, demonstrating that their interaction is compatible with the tRNA aminoacylation activity, and supporting the possibility that the loss of one tRNA binding surface allowed SLIMP/mSerRSα to establish new functional interactions (Figure 5).

## DISCUSSION

In animal mitochondria, two different tRNA^Ser^ forms co-exist: the near-canonical tRNA^Ser^_UGA_, and the highly diverged and structurally distinct tRNA^Ser^_GCU_. The realization that mitochondrial SerRS2 in *B. taurus* was capable of efficiently aminoacylating these two very different substrates (22) prompted an investigation on the molecular features of SerRS2 that permit such substrate diversity. Studies on *Hs*SerRS2 revealed an adaptive mechanism that allows the enzyme's tRNA binding surface to recognize its two highly divergent cognate substrates (17).

In *Drosophila*, the aminoacylation of mt-tRNA^Ser^ is performed by *Dm*SLIMP/*Dm*SerRS2α, a heterodimeric SerRS2 composed of the monomers *Dm*SLIMP and *Dm*SerRS2α (23,25). How *Dm*SLIMP/*Dm*SerRS2α evolved, and how it recognizes tRNA was not understood. Here, we set out to investigate the evolutionary history of animal mitochondrial SerRS2, and the structural and functional diversity of this group of enzymes. To achieve this aim we determined the phylogenetic relationships of animal SerRSs and characterized the structural and functional differences of their mitochondrial forms.

The phylogenetic analyses presented here show that the SerRS2 duplication that gave rise to SLIMP took place at the root of bilaterian evolution (Figure 1A, B). The duplicated gene that would eventually become SLIMP was fixed in all insect, echinoderm, hemichordate, mollusc, annelid, and tardigrade species for which complete genome information is available, where it is always accompanied by an additional SerRS2 sequence (SerRS2α). Distance-based phylogenetic trees clearly show that the catalytic domain sequences of SLIMP evolve at a faster rate than SerRS2α sequences (Supplementary Figure S1).

**Figure 6.**
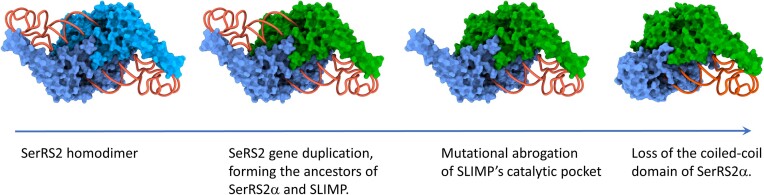
Proposed structural and functional evolutionary history of metazoan SerRS2.

The structural analyses based on the heterodimeric *Dm*SLIMP/*Dm*SerRS2α model reveal the same process of sequence divergence and domain loss in all SLIMP-containing species (Figure 2A) involving the mutational collapse of the catalytic pocket of SLIMP, and the disappearance of the tRNA-binding N-terminal coiled-coil domain in SerRS2α. Possibly, the formation of non-productive complexes after the collapse of the active site in SLIMP was a factor in the selection of a SeRS2a variant devoid of the coiled-coil domain. These changes led to the complete loss of one of the two active sites found in homodimeric SerRSs, highlighting how gene duplication events can lead to the separation of molecular functions within a protein complex (Figure 6).

Sequence comparisons show that the process of asymmetrical domain loss in SerRS2 extends to most invertebrate species (Figures 2B, C). However, echinoderm species such as *S. purpuratus* and *A. planci* show an apparently higher conservation of canonical SerRS structures. The apparent retention of a coiled-coil structure by *S. purpuratus* SerRS2α suggests that the mutational collapse of the catalytic pocket in SLIMP may have preceded the loss of the coiled-coil domain in SerRS2α (Figure 6). Considering that Echinodermata represent the closest sister clade to chordates (where SLIMP was not retained), it is possible that the evolution of the SLIMP/SerRS2α heterodimer was slower in deuterostomes (echinodermata/chordata), and was later fully reversed to a canonical SerRS2 structure in chordates alone.

The comparison of all metazoan SerRS2 sequences also reveals that, in the nematode species analysed, SerRS2s also lost the coiled coil domain (Supplementary Figure S1). Interestingly, however, we could not detect SLIMP in these nematode species, indicating that nematode SerRS2 followed a different, and as yet uncharacterized, evolutionary path.

In *Drosophila* the evolution of *heterodimeric Dm*SLIMP/*Dm*SerRS2α led to the acquisition by this enzyme of additional interactions and regulatory functions (25), but these did not affect the aminoacylation function of the heterodimer, as evidenced by our experiments using LON domains (Figure 5). Our data indicate that a heterodimeric SerRS2 composed of SerRS2α and SLIMP is probably the established functional enzyme in all protostomes, but it remains to be determined if the non-canonical functions of *Dm*SLIMP/*Dm*SerRS2α extend to other SerRS2 enzymes in SLIMP-containing species.

Our mutational data indicate that the tRNA recognition mechanism used by the single active site surface of heterodimeric SerRS2 is equivalent to that used by mammalian homodimeric SerRS2 (Figures 4C, D). This tight conservation of the tRNA recognition mechanism is compatible with the large structural variations in mt-tRNA^Ser^_GCU_ and tRNA^Ser^_UGA_ isoacceptors, as previously noted in the analysis of the tRNA recognition modes of human SerRS2 (17). Thus, although the degeneration of mitochondrial tRNA structures may be an consequence of Muller's ratchet (44,45), the sequence and structural drift seen in tRNA^Ser^ preserves the interaction with SerRS2, reinforcing the idea that tRNA molecules are fixed by their complex set of interactions and are only free to evolve in simplified scenarios such as the mitochondria, and only in directions permitted by their remaining functional constraints (46,47).

The natural history of animal mitochondrial SerRS illustrates how central to the evolution of this molecular system is the conservation of tRNA recognition and aminoacylation mechanisms, which impedes the free drift of all structures involved. However, peripheral regions of the system, be it the tRNA structure or the aaRS architecture, are under lighter selective pressure and can evolve as long as tRNA charging activity is not compromised. As result of this uneven selective pressure, large variations in specific structural regions of mt-tRNA^Ser^ and SerRS2 appeared and evolved to acquire new interactors and functions in invertebrates. Interestingly, vertebrate SerRS2 reverted this evolution and returned to the ancestral homodimeric state, through a process whose understanding will likely require characterizing SerRS2 in echinoderms.

## Supplementary Material

gkad696_Supplemental_File

## Data Availability

All supporting data used in this article have been deposited in a public repository, and can be found at the following DOI: https://doi.org/10.6084/m9.figshare.23560404.v1.

## Notes

Present address: Hubrecht Institute for Developmental Biology and Stem Cell Research, Protein structure, mechanism and design, Utrecht, The Netherlands.
