# Supplementary material for: Domain collapse and active site ablation generate a widespread animal mitochondrial seryl-tRNA synthetase
Source: Nucleic Acids Res. 2023 Aug 28;51(18):10001–10. doi: 10.1093/nar/gkad696 (PMC10570016; doi:10.1093/nar/gkad696)
Supplement: gkad696_Supplemental_File [file gkad696_supplemental_file.pdf]

Supplementary figure 1. Phylogenetic analysis of metazoan SerRS, SerRS2, SLIMP, and SerRS2α sequences.

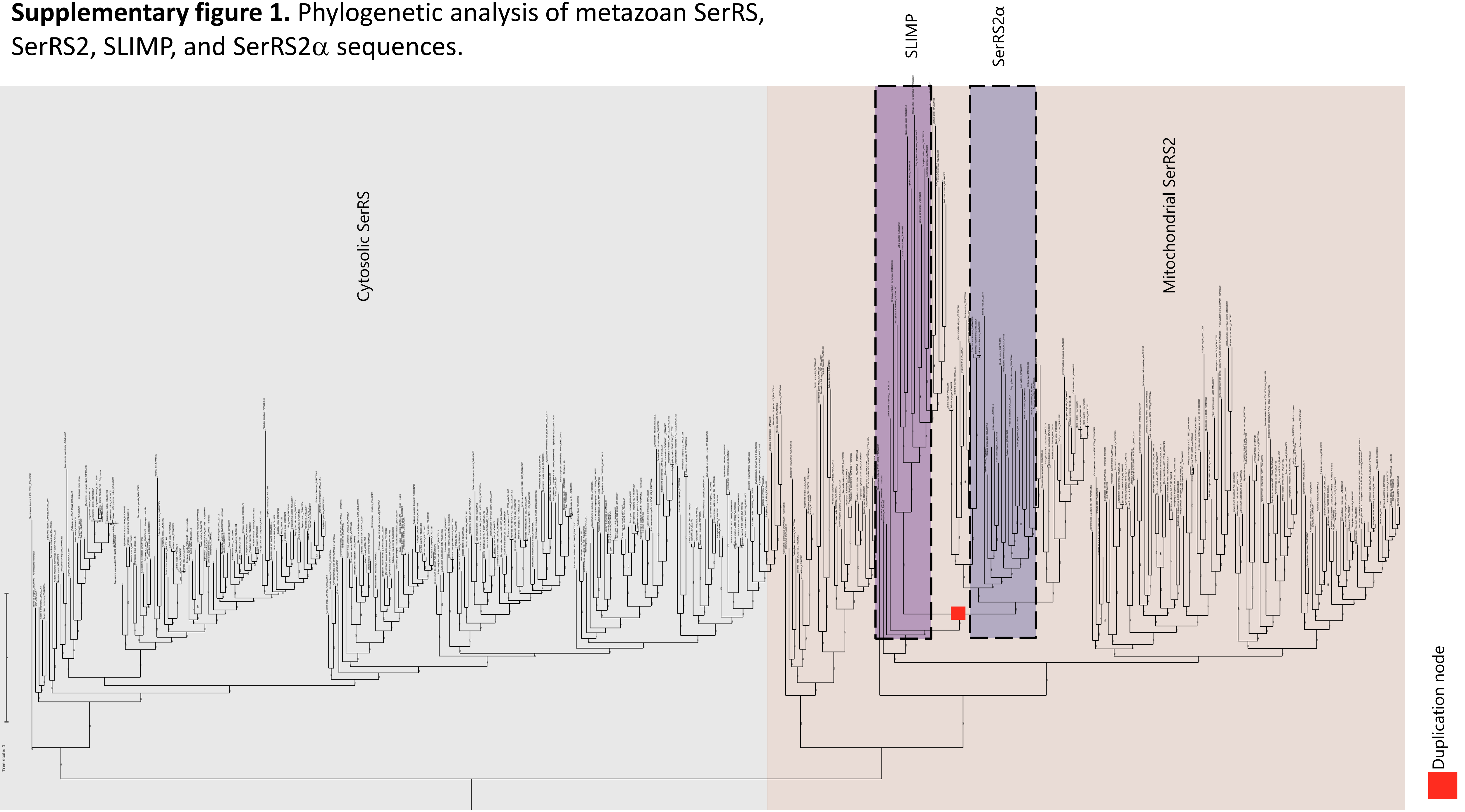

Supplementary Figure 2

```
Drosophila_melanogaster_SLIMP             LSKVAEERERVTKRL----KELTKS---GSSAVQLEELKEHGKSLRNELKALKQTLYPIE     137
Acanthaster_planci_SLIMP                  KSCIEDRRSKVNEDI----KRLRKK---GGAKEQKDKLQQMGKSLREELRQINFSIASLS     196
Strongylocentrotus_purpuratus_SLIMP       KNQMERKKKALNTRA----KELSKQ---KGGSVTKEKVQLEGRKIREEVKSLNTSLNGLM     191
Triboleum_casteum_SLIMP                   KTVLEFTRSEIQNLI----NKLKQDGEE-KNQSDIEKLLLHIKVVKDDLKNVKEFTYGLE     133
Stegodyphus_mimosarum_SLIMP               YQEIVEKEEEYQKEL----ERLQSLDL--ET--EIKDITEKRIALSQNLRLEKEKLYDVE     156
Ramazzottius_varieronatus_SLIMP           RAELETRRKQSVENF-------------SKPSDVDEESKLKGRLLRGEIKQLNKDIAEIE     168
Pediculus_humanus_SLIMP                   KLQLEKRKTQIADNI----KDLKKIGNDGEAKQEMEKLKIEGRIVREDLKTLMKAFWEVE     140
Octopus_bimaculoides_SLIMP                KKKLEEERKNIASMM----AALSKVKETEDVKRSKELLKSRGKSLRQLLKTLNADFNILE     178
Limulus_polyphemus_SLIMP                  LQELLRRKENISQKL----QELSKTNH--ITSEEKENWKVEGRKVREEIKHVTEKMWDYE     160
Lottia_gigantea_SLIMP                     KCDFESERKDIGKRVTELINLMKKSGENDNLVKSVDVLKSRGNKLKKLEKELSDRYFAIK     173
Hyalella_azteca_SLIMP                     IEELEKQADELTVTS----NEMK-----GKEDVKDDEVKVRISQLRSKTKILKARLNELE     347
Daphnia_pulex_SLIMP                       REDLETRRIEVTQLM----KQVASHTADTTRPNKIEEVKQEGIRLRTQLKELTKVWWDVE     63
Capitella_teleta_SLIMP                    KDLLEKQHKEISRGFAELVRAKK---NDDIAKEAKKELSEKGRELRPRLKAAITSYYEAE     226
Anopheles_gambiae_SLIMP                   KKEIEQYRVDLQKRI----Q----Q---SSSKEEQQQLKSRAVLARDDLKMLKEQSYKVA     127
Drosophila_melanogaster_SerRS2a           ---------------------------------------------------------HKL     75
Acanthaster_planci_SRS2a                  LNDLQDEG--------------------------------SSPALANHLDLLKTQIRETE     148
Strongylocentrotus_purpuratus_SerRS2a     VHDVQLRLQTDIDK------------------------GLNTKELESMLKEKKNQLDRAQ     160
Triboleum_casteum_SerRS2a                 ----------------------------------------------------DKLYNATK     82
Stegodyphus_mimosarum_SerRS2a             --D-------------------------------------------------IEKCQILK     33
Ramazzottius_varieronatus_SerRS2a         ------------------------------------------------------------     0
Pediculus_humanus_SerRS2a                 -----------------------------------------------------GKRETLW     92
Octopus_bimaculoides_SerRS2               -----------------------------------------------------MKKQGLI     113
Limulus_polyphemus_SerRS2a                --E-------------------------------------------------ETERALLE     99
Lottia_gigantea_SerRS2a                   --A-------------------------------------------------SHK-DRIW     113
Hyalella_azteca_SerRS2a                   -------------------------------------------------SLNSVTSEDEL     87
Daphnia_pulex_SerRS2a                     ----------------------------------------------------SADQMELK     82
Capitella_teleta_SerRS2a                  SDR-------------------------------------------------SEEFMSLQ     90
Anopheles_gambiae_SerRS2a                 ----------------------------------------------------ESERGTLQ     83
Homo_sapiens_SerRS2                       IRSLEEEKAAVTEAVRALLANQDSGEV--QQDPKYQGLRARGREIRKELVHLYPREAQLE     160
Bos_taurus_SerRS2                         IRSLEEEKEAVTEAVRALVVNQDNSQV--QQDPQYQSLRARGREIRKQLTLLYPKEAQLE     160
Danio_rerio_SerRS2                        ISHLEGRKQVVSSTVRELVGKHDKSTL--SSLEEYGHAREEGRAIREKLNQLYLQEKELE     186
Xenopus_tropicalus_SerRS2                 IKTLEEEKSAIAKEVKHMVISEEKQNL--QTNPRYALIRKRGKDIRLELSTLYKEESNLE     233
Caenorhabditis_elegans_SerRS2             GEK-----Q-------------------------------------TNGAVSEQKYKQLW     106
Trichinella_spiralis_SerRS2               KNP----------------------------------------------TMTMNELTSMW     82
```

Full-length sequence alignment of SerRS2, SLIMP, and SerRS2α metazoan sequences (labeled accordingly).

Supplementary Figure 2

```
Drosophila_melanogaster_SLIMP           DDFIH-DYLHLPNLLHVQCPVGGEEK-------LLYRHGIP-K-------S---------     172
Acanthaster_planci_SLIMP                DEFYP-AVLKLPNKLHPAVSLFFQTQPCGSMLKILETFGTD-K-EEKSDKP---------     244
Strongylocentrotus_purpuratus_SLIMP     NRLYP-LALALPNSLHNAVPVG-------DENVVKETVGDG-L-VSLE------------     229
Triboleum_casteum_SLIMP                 ENANL-GVLSLPNFLHPKTPLDSQTI-------I-HEFLEQ-P-------K---------     167
Stegodyphus_mimosarum_SLIMP             DIVMP-KLQKIPNLIDAATPTQFDT----------ADICQK-S-PDFEFKV---------     194
Ramazzottius_varieronatus_SLIMP         IDTIT-TLLKLPVRLHKYVPMGNNPF-------VSFDTTAD-QEVQFMECSKLNSNSVNA     219
Pediculus_humanus_SLIMP                 EKVIM-KGLSLPNELHKDTPDGEDKI-------V-HQYKDR-P-------I---------     174
Octopus_bimaculoides_SLIMP              ETVIM-RSLKLPNVLHRDTPSDEVKI-------LQETVLEK-DLADFA------------     217
Limulus_polyphemus_SLIMP                DDAIP-VLLSIPNNIHPDTPLQDDDI-------I-GNFCSN-N-QDNCIQN---------     200
Lottia_gigantea_SLIMP                   HAVML-DGLKIPNRIHPETPTSAQIL-------RSF----G-NIMDFSK-----------     209
Hyalella_azteca_SLIMP                   VTAIP-AMLDLPNTVNLPPGDGGVV----------VSLNDP-PIFDFSPKP---------     386
Daphnia_pulex_SLIMP                     ETAVT-RALSLPNSLHSETPIEDSRE-------L-YSFIPP-K-------S---------     97
Capitella_teleta_SLIMP                  NTFNL-MALKLPVELAPETQPDTSTL---------AELGVK-AAES--QKSHLEL--L-G     270
Anopheles_gambiae_SLIMP                 DAFIANSFLSIPNDLHERTPEEGAKC-------I-YEKPVP-P-------R---------     162
Drosophila_melanogaster_SerRS2a         RQLQA-ELEQLPNATHPRLR-DYGEE-----PRELAQYVHRQLTPQSRLKEFSELAR---     125
Acanthaster_planci_SRS2a                DVFYQ-SAARLPNRTHPEVPMGGGDE-----PRLVMLIGEK-PSFNFPIKSHVELGE---     198
Strongylocentrotus_purpuratus_SerRS2a   NDFYR-IAVAIPNSIHPDVPLTEDAD-----PVLIEEINEK-RSFDFPIKGHVELGE---     210
Triboleum_casteum_SerRS2a               SEFET-EVLRIPNRTHPQVA-GYGDS-----PKTVEIVGT--KPPDSSHLDFQEICK---     130
Stegodyphus_mimosarum_SerRS2a           EKIAP-QLLWFPNLTHPKVLENKSEE-----PFLLQKVGKK-TAFTYFPTKFESLTK---     83
Ramazzottius_varieronatus_SerRS2a       -------------------------M-----AAAVEVIGTK-QAFRFAPKSIETLAA---     26
Pediculus_humanus_SerRS2a               QSLVK-EAGSLPNKTHPAVK-EYGER-----PRVLKSSEH--VTHSFIPKDFQELAK---     140
Octopus_bimaculoides_SerRS2             DKLKS-LAADIPNKIHPLAPIGNESC-----NRVVKTFGKE-SVNVGKVKGVVEIGE---     163
Limulus_polyphemus_SerRS2a              EKLIS-EAAKLPNKTHPKVLENSSDQ-----PVEVETVGSA-PVFDFKPKQFHELAL---     149
Lottia_gigantea_SerRS2a                 KDLIK-EASDIPNTHHPDSPIGEEEN-----SRLVETVGEP-TRFNFKPRTVVRIGE---     163
Hyalella_azteca_SerRS2a                 INMYK-NLSKLPNATCPIAE--ELSG-----PKIIGLFGTKRVLDDALML--EQLSH---     134
Daphnia_pulex_SerRS2a                   KTLLA-EALKIPNNSHPRLH-AYGDA-----PHVVETPGEK-PNWSFKPLEFHSIAK---     131
Capitella_teleta_SerRS2a                EKFHK-SAGVIPNMSHPDAPIGGEDK-----AILVETFGEK-QDFDFAPKGVVEIGE---     140
Anopheles_gambiae_SerRS2a               AQLDA-ELAKLPNRTHPAVV-GYRTE-----PRIIERYNEHAKRTDRKYFQFGDICK---     133
Homo_sapiens_SerRS2                     EQFYL-QALKLPNQTHPDVPVGDESQ-----ARVLHMVGDK-PVFSFQPRGHLEIGE---     210
Bos_taurus_SerRS2                       EQFYL-RALRLPNQTHPDVPVGDESQ-----ARVLHVVGDK-PAFSFQPRGHLEIAE---     210
Danio_rerio_SerRS2                      KEHYC-RALRLPNRTHPSVPIGDESQ-----ARVVEVVGEK-REFDFKPKGHLQIGE---     236
Xenopus_tropicalus_SerRS2               GSFYP-RALRLPNRTHPNTPVGDESK-----ARVLEVVGVK-PEFDFKICGHLEIGE---     283
Caenorhabditis_elegans_SerRS2           DELYD-EAILIPNMTQDGVPRGSEDQ-----AKIVAEWGEKREDECLTAEKLVQ------     154
Trichinella_spiralis_SerRS2             DRFYE-EALLIPNDTHPEAPVGDESV-----AKIIHTNGSK-PIFTFKPLFGEDIAV---     132
```

Supplementary Figure 2

Drosophila_melanogaster_SLIMP	------------ENKTTS----HLARQELVHFVDN**N**RYYLMEQAALFDVNAMQSLARYFV	216
Acanthaster_planci_SLIMP	----------------KEQS-PNIFLTESHPMSHG**G**YAYLEHEAALLELALTQ-WVKQQL	286
Strongylocentrotus_purpuratus_SLIMP	-----------------SRK-EHPSISTHHPMVHP**G**YCYTEGELVIKELDLVL-SAASHL	270
Triboleum_casteum_SLIMP	------------PIQ-ENHL-QIAKSQNLIEYTSP**T**LCYLKSDAALFEFAITN-YFQSNL	212
Stegodyphus_mimosarum_SLIMP	--------------ISHAEL-GKLMSLLEFSNNDP**T**VYYMKGKLSELELATQI-YFSDKL	238
Ramazzottius_varieronatus_SLIMP	ANRVARREKVRVEAHDVNKA-LQQSRLVEYTDEAP**R**GYFLRGQAAELELKLLN-YGRSFL	277
Pediculus_humanus_SLIMP	------------INQKYSHL-DIGQKQNLIDYRDP**S**LCYLKSEAALFELATSS-YFSEGL	220
Octopus_bimaculoides_SLIMP	-------------QVNHVVA-GEQYNWIKFSNIGP**G**AYFLLGPLAWSERHFIN-HFSENL	262
Limulus_polyphemus_SLIMP	------------LKACRSHL-QVLPNDIQFSNVSP**C**SYYLTRNAALLELALIY-FFSHHL	246
Lottia_gigantea_SLIMP	-------------SRSHIEI-GREKDLIKFSDIGP**K**SFYLKGDLALCELELID-KTKKFL	254
Hyalella_azteca_SLIMP	------------H---TLLR-TQSPNSLEFLDVSP**G**AFYLSGDLAECEVLLDR-YWTGLL	429
Daphnia_pulex_SLIMP	------------AHS---PS-DSTKLDVKFVSHSP**T**AFYMRGLSARLELNWMR-NFSNDW	140
Capitella_teleta_SLIMP	GSGIH----LRYV---FPAS-SPHINTPCPSDISP**G**AYYLTGNVSNSERQLVQ-WTKKFM	321
Anopheles_gambiae_SLIMP	------------AATSNRPS-NVDPVQEQLEEFGP**A**CLYMTGDAAWMDLRLPM-YCSELF	208
Drosophila_melanogaster_SerRS2a	------------------AL-NLYRMDHLGNYTGH**K**SYYLTGQLATLEQAIIQ-YALQAV	165
Acanthaster_planci_SRS2a	------------------HL-DIIRIKNLGHVTGH**R**SYFFKGAAAMLEHALIC-FTVDRL	238
Strongylocentrotus_purpuratus_SerRS2a	------------------DL-DIIRLKNLGHTTGH**R**SYFLKGAGSMLEDALVR-FTLDRL	250
Triboleum_casteum_SerRS2a	------------------KL-RLLRTDQIGNFCGS**K**SYYFLGELAELEHALVR-YVTSKL	170
Stegodyphus_mimosarum_SerRS2a	------------------RL-NVLRTKHLGHMSGS**K**SYFLKNDLSLLEQALVR-FALSKL	123
Ramazzottius_varieronatus_SerRS2a	------------------GYAGSLRRTKLSLLSTD**R**AYILGGLFAKLEQALIS-FTTGFL	67
Pediculus_humanus_SerRS2a	------------------DL-KLLRMEDLTTVAGQ**R**SYFFTGDLADLEQALIK-YTISRL	180
Octopus_bimaculoides_SerRS2	------------------KA-GWLRTSNVGTTTGQ**R**SYYFLNQLARLELALVN-YCLDKL	203
Limulus_polyphemus_SerRS2a	------------------KL-DMLRMENLGLVTGH**R**SYYLKRELAQLEQALLH-FSVKRL	189
Lottia_gigantea_SerRS2a	------------------KL-DILRTDNVNFSTGI**R**TYYFKDELVDLEMALIK-FTLNTL	203
Hyalella_azteca_SerRS2a	---------------------HGVDMNMTGNFTGS**R**SYYLRGSMAHLESKLTQ-YALEHL	172
Daphnia_pulex_SerRS2a	------------------DL-DLLRTENLGNLTGP**R**SYYLIKELAQLEQALIH-FTVDAL	171
Capitella_teleta_SerRS2a	------------------KL-GVLRTQNVTYTTGP**R**SYYFMGDLAELEDALVE-FTISRL	180
Anopheles_gambiae_SerRS2a	------------------RM-NLYRMENLGNFTGH**R**SYYLTDELAELEHALIC-YAVDSL	173
Homo_sapiens_SerRS2	------------------KL-DIIRQKRLSHVSGH**R**SYYLRGAGALLQHGLVN-FTFNKL	250
Bos_taurus_SerRS2	------------------KL-DIIRQKRLSHVSGH**R**SYYLRGAGALLQHGLVN-FTLNKL	250
Danio_rerio_SerRS2	------------------SL-DIIRQRRLSHVSGH**R**SYYLRGAGAQLQFALQN-FAMDLL	276
Xenopus_tropicalus_SerRS2	------------------DL-DIIRQRRLSHVSGH**R**SYYLRGAGSLLQHALVN-FTISKL	323
Caenorhabditis_elegans_SerRS2	---------------------TWRSLLHPTDASGQ**R**SYVFLGALASLEKALLD-YAHERV	192
Trichinella_spiralis_SerRS2	------------------KF-KIL-WQDVGFCCGE**K**AYFLYNQLAILELALIR-FCRDRL	171

Supplementary Figure 2

```
Drosophila_melanogaster_SLIMP              N-HGHFIQTANPDFVRCVLL---EANATPL--SDYHLVQE------EHL-----------     253
Acanthaster_planci_SLIMP                   L-DQGFTPLSCPAMYKSIAL---ESLGLDP--DNSNETYAIET-----------------     323
Strongylocentrotus_purpuratus_SLIMP        T-SNGFTRFAMPDMYKPLLR---EALGLNA--EEFNETYAIQT-----------------     307
Triboleum_casteum_SLIMP                    V-SQSFTQFSNADFVRSLVV---EGCGTDY--LDKNKIFTLDGS--NNE-----------     253
Stegodyphus_mimosarum_SLIMP                I-SLSYAPLSCVDFCKSFII---EAVGLDP--HSNADCIPLKS---KSS-----------     278
Ramazzottius_varieronatus_SLIMP            E-EKGYSGISGPHLYRSVVV---EGTGFIS--DDHDDVSATTGSLINVKPNQPFLIDTVD     331
Pediculus_humanus_SLIMP                    K-ADEYIPFSNPDFVRSLVV---EGCGADH--SDPNSIFTVLLP--NEK-----------     261
Octopus_bimaculoides_SLIMP                 I-QNGFQMMVCPEIFKAVVV---EGCGVDI--TDTGKIYTLEN---NEE-----------     302
Limulus_polyphemus_SLIMP                   A-DAGLTEISCPDIVKSVIV---EGCGNSF--MSSIS---------LIS-----------     280
Lottia_gigantea_SLIMP                      Q-QNGMTTFLLPDKVRSIIM---EGCGFDV--DSDKILTIHDP---DPR-----------     294
Hyalella_azteca_SLIMP                      D-AAGYLRQCNPDFAKEVLV---EGCGGAS--SLKTQL----------K-----------     462
Daphnia_pulex_SLIMP                        I-SKGYNLISPPDFVRSLIV---DGCGLDF--NNPQKVLSLATVPDHGS-----------     183
Capitella_teleta_SLIMP                     N-DLSIPRISCPELFREAIV---EGCGFTD--EEHISHAC--------------------     355
Anopheles_gambiae_SLIMP                    R-RNKCILFCNPDFVRSFLV---EAAMVSK--ESLFLVQE-----EDEP-----------     246
Drosophila_melanogaster_SerRS2a            T-EHGFKLISVPDILPKEVI---ESCGMRT-EGERTQVYKLDT-----------------     203
Acanthaster_planci_SRS2a                   L-GRGFRAISVPDLVHPIVF---EGCGMRT-KGLHTQVYQLDHRKH--------------     279
Strongylocentrotus_purpuratus_SerRS2a      IKNHGFKIIKVPDLIKPVVFVSIEACGMRT-EGLHTQVYPLSPEYH--------------     295
Triboleum_casteum_SerRS2a                  L-FNKFELVSVPDILPRGTI---EGCGMNT-KGARTQVYALDSTLH--------------     211
Stegodyphus_mimosarum_SerRS2a              I-KKGFQMVSVPDVVSQTSF---EGCGMKT-DGKHSQVYHIEDSWA--------------     164
Ramazzottius_varieronatus_SerRS2a          K-QRGFEFISVPDIISPEVL---DGCGMERLKGRHNMIYLLDEQRH--------------     109
Pediculus_humanus_SerRS2a                  L-KKNFQLISVPDILPMEVL---ERCGMTP-NALQTQVYQLDES-H--------------     220
Octopus_bimaculoides_SerRS2                R-LEGFQLVSVPEILRPEII---EGCGFKT-TGQRTQVYKLDAKRH--------------     244
Limulus_polyphemus_SerRS2a                 M-SKGFTLVSVPDILHPSAI---EACGMTT-QGKRTQVYHLEPNFG--------------     230
Lottia_gigantea_SerRS2a                    K-QHGFHVISVPDLIYAPVI---EACGFKT--TGENQVYKLNDNFY--------------     243
Hyalella_azteca_SerRS2a                    K-RKGFKLMSVPDLVTEDIL---ESCGVPV-HGERTMVYSVSPSHLGLP----------S     217
Daphnia_pulex_SerRS2a                      E-KKGFILYSVPDLLHSRLI---ESCGMDT-KSERTQVYKLEPKFY--------------     212
Capitella_teleta_SerRS2a                   Q-AMGFCVISVPNLLHSSVI---EGCGFQV-QGERTQVYRSQ--DD--------------     219
Anopheles_gambiae_SerRS2a                  R-QRNFQLVTVPDVLPGRII---ESCGMSV-HGERNQVYKLHGRDG--------------     214
Homo_sapiens_SerRS2                        L-RRGFTPMTVPDLLRGAVF---EGCGMTP-NANPSQIYNIDPARF--------------     291
Bos_taurus_SerRS2                          I-HRGFTPMTVPDLLRGVVF---EGCGMTP-NAKPSQIYNIDPSRF--------------     291
Danio_rerio_SerRS2                         Q-KRGFIPMVVPDILKSVVF---EGCGMQP-HAQKSQVYSLNPKRS--------------     317
Xenopus_tropicalus_SerRS2                  V-KKGFIPMSVPDMLRGAVF---EGCGMQA-DAHASQVYSVDPLQH--------------     364
Caenorhabditis_elegans_SerRS2              C-ALGFRPITVPDIVSGEVT---QACGVMQ-RSDHPIQYTLAGEES--------------     233
Trichinella_spiralis_SerRS2                R-SSGYEEVVVPDVIPKRIV---ENCGLMN-RTDKEIVYSIHGL----------------     210
```

Supplementary Figure 2

```
Drosophila_melanogaster_SLIMP          -QNKINTAYLTGGASFESYLGAMTKLCVYPS------------VLPLRYVCCGRSYNRAE     300
Acanthaster_planci_SLIMP               ----HENVQYLAGVSSLAFVAYFMMSVVEAS------------DLPYRCFTVGRSYKQHQ     367
Strongylocentrotus_purpuratus_SLIMP    ----RENLMHLAGTSPMGFLAYYMNSVLEAS------------DLPQRCFAVGRHYNAAA     351
Triboleum_casteum_SLIMP                ----INRLHLVGGASLYSFMAYFAKNSIQSA------------LLPLKFFTLGRKYSPGS     297
Stegodyphus_mimosarum_SLIMP            -RESGQKLNLVGGASLEMFCAYLTNMNVDKS------------SFPLRYYSLGRRYNAQN     325
Ramazzottius_varieronatus_SLIMP        TRGSLFRSLFLTNSTVNLYAAHFTNCEAAEN------------ILPIRLHSSSRVYRAVS     379
Pediculus_humanus_SLIMP                -VQPNYRLHLVGGASIYPFCAFHTKYTIGFS------------SLPLKYFTVGRQYKPTL     308
Octopus_bimaculoides_SLIMP             -TMKIEHICHFIGVSPMSFAAFWTRMTVDCE------------ATPQTYFTIGKCYHPVV     349
Limulus_polyphemus_SLIMP               -EQKGAMSILTGGASFESLIAYFTRSIVEKT------------SLPLTFFSVGRCYQSLN     327
Lottia_gigantea_SLIMP                  -ESKVIDEHHLQGNSVQSFVAFLMRQMVDNS------------QLPLKYFSVGSRYVSG-     340
Hyalella_azteca_SLIMP                  -EEDKTPVYLVGGASLPAYLGYFARRAVKKT----------SKFLPQKLYACQRTYTPLR     511
Daphnia_pulex_SLIMP                    -LEKGNGFHLVGGSSLPAMVAFLTKNAIEE-------------PIPLRLASAGRTYHPPT     229
Capitella_teleta_SLIMP                 ---------------------IFMRLIFPFN------------ATHRR------------     370
Anopheles_gambiae_SLIMP                -VDKVNLLHLCGGGSLLSFLGYFTKLSVFPS------------ALPLRLVASGKRYHFE-     292
Drosophila_melanogaster_SerRS2a        -------GECLSGTSEMALAGFFANKLLSED------------QLPLKVTAVSRCYRAET     244
Acanthaster_planci_SRS2a               -----NMDLCLSGTAEVGIAGYFMDTVLDVA------------ELPQKKFAISQCFRAET     322
Strongylocentrotus_purpuratus_SerRS2a  -----NSDLWLAGTAEVGIAGYLMNKTIPMA------------ELPLRIAAVSRCYRAET     338
Triboleum_casteum_SerRS2a              -----GPDWCLSGTSEMALAGYLSNKTLNSD------------ILPLKLAAVSRCYRAET     254
Stegodyphus_mimosarum_SerRS2a          ------PRLCLSGTSEIALASHFSQKTFQNS------------NLPLKLCAASRCYRAEA     206
Ramazzottius_varieronatus_SerRS2a      ------GRFALSGTSEMAIAALHRNKIIHRD------------QLPIKLSAMSRCYRAEV     151
Pediculus_humanus_SerRS2a              -----GQNIALSGTSEMALAAMNSNVVFNLK------------DLPVNLAAVSRCFRSEV     263
Octopus_bimaculoides_SerRS2            ------GSACLSGTSEMALAGYFQNQEFSLQ------------DLPIKLTAVSRCHRAEI     286
Limulus_polyphemus_SerRS2a             ------GDLCLSGTAEMAFGAFFANKTLSLN------------ELPQKLAAVSRCFRAET     272
Lottia_gigantea_SerRS2a                ------QDVCLAGTAEMSLAGFMSDEKLTTE------------NLPTKLTAVSRCFRREA     285
Hyalella_azteca_SerRS2a                PSKEESKKVCLSGTSEMALGGWLRNHSFTEE------------QLPLKLCAVSRCYRAET     265
Daphnia_pulex_SerRS2a                  ------GDICLSGTAEMALASYFTGSAVPVS------------ELPIKMAAVSRCYRAET     254
Capitella_teleta_SerRS2a               ------PSVCLAGTGEMPLAGYFMNKQFDIK------------DLPQRVATVSRCYRAET     261
Anopheles_gambiae_SerRS2a              -----REPLCLSGTSEMALAGYLAGRVLPAD------------RLPQKLMAVSRCYRAET     257
Homo_sapiens_SerRS2                    ------KDLNLAGTAEVGLAGYFMDHTVAFR------------DLPVRMVCSSTCYRAET     333
Bos_taurus_SerRS2                      ------EDLNLAGTAEVGLAGYFMDHSVAFR------------DLPI-----STCYRAET     328
Danio_rerio_SerRS2                     ------PDLNLAGTGEVGVAGYFMDHAVNFK------------DLPVRTVCSSTCYRAET     359
Xenopus_tropicalus_SerRS2              ------PDLNLAGTSEVGIAGFFMDHAVMLQ------------DLPVRTVCCSTCYRAET     406
Caenorhabditis_elegans_SerRS2          -------HTKLSGTAEMGIAAFLRGRTFNQD------------QLPIRLVSLSRCFRTEI     274
Trichinella_spiralis_SerRS2            ------PNWTLSGTAEMGLASFIENRIFDVN------------EMPKKFFSVSRCFRPEV     252
```

Supplementary Figure 2

```
Drosophila_melanogaster_SLIMP          ADL--------------YGPIPSLYTATQTNAVQIFVATQTD--NEADSQLEHILNLATD     344
Acanthaster_planci_SLIMP               ES----------------TYFEGLQESFQSQRVELFGVCENT-EAASNALFNQFLSTARG     410
Strongylocentrotus_purpuratus_SLIMP    ES----------------AKFQGLHHQYQDSRLEIFGVSEGA-EDSSNALLFQYLHIVTD     394
Triboleum_casteum_SLIMP                G--------------------PDLLNLSQNTVGQIFVATSNN-SNE---IFDSLVQQIRE     333
Stegodyphus_mimosarum_SLIMP            -----------------RVNSQDLFSVVQSTNIHSVILCDQN---SEKEEFSAFFNSVLL     365
Ramazzottius_varieronatus_SLIMP        NK-----GLEEEE----DEKYCRIFRPSQTHTVSFCTAFTDA--ETTEEYLTETLELFKS     428
Pediculus_humanus_SLIMP                QEQ-------------DDESSSGLFNLWQRTCVDMFALTADN-YNEMMSIFGKTLNIVIN     354
Octopus_bimaculoides_SLIMP             SS----------------CANMGLYNTSQTTKCEVCSVCHDD--TMADNQFQQFLTMIWN     391
Limulus_polyphemus_SLIMP               PQ---------------QLTIPGLFSVPQRTEIHFIIACEES---NEKENFGKLVTLLQQ     369
Lottia_gigantea_SLIMP                  ------------------TELPGLFGGHQYANAVIFGASLSE--TESSNLFQEYSELIWS     380
Hyalella_azteca_SLIMP                  DLCPPDRGLHAPHEGLYAPQMRGVMSSMQSTSVSLLTMYTNE-SKFKDLVKDEVLGVVVQ     570
Daphnia_pulex_SLIMP                    ESN-------------HE----DLTNTTQASSIHLLTVMKNC-PDAMFKEVVRVQETISM     271
Capitella_teleta_SLIMP                 ---------------------------WFTLQVDMFSLNKSK--DESSLSMAELAFNITQ     401
Anopheles_gambiae_SLIMP                --------------------------SAQSNEVQVFGAHTTA--QGAVELFDEMVQIYQQ     324
Drosophila_melanogaster_SerRS2a        SGL---------------QEEKGIYRVHQFNKVEMFAICTEE---QSEAELEEFKNIEVD     286
Acanthaster_planci_SRS2a               THS---------------AEARGLYRVHQFMKVEMFCVATED---QSEYIFDELIAIQRD     364
Strongylocentrotus_purpuratus_SerRS2a  AHS---------------ADARGLYRVHQFTKVEMFCVAANESGKESEQIHQELVGIERE     383
Triboleum_casteum_SerRS2a              SNL---------------LEERGIYRVHEFTKVEMFVVTQPQ---NSEQALEDILKLEMD     296
Stegodyphus_mimosarum_SerRS2a          NKH---------------QKEKGIFRVHHFTKVEMFGITANDSGNESEELLEEFTELQKE     251
Ramazzottius_varieronatus_SerRS2a      SDV---------------Q--EGIYRVHEFTKVEMFGLCQPD---DSKTMLEEFVTIQKD     191
Pediculus_humanus_SerRS2a              SHF---------------AEEKGLYRVHQFTKVEMFGISHPD---NSEEMMQNFVDIQEE     305
Octopus_bimaculoides_SerRS2            SEL---------------EEEKGIYRVHNFTKVEMFSVVAQETGKESDDSLQEFIDLQTD     331
Limulus_polyphemus_SerRS2a             STV---------------QEEKGIFRVHQFTKLEMFGVTANETSTESEQLLDEFTTIQRS     317
Lottia_gigantea_SerRS2a                SDA---------------KEEQGIYRVHQFTKIEMFGVTENDKGTESEELLQHFLDIEKS     330
Hyalella_azteca_SerRS2a                SKL---------------KLEQGIFRVHHFTKVEMFGITSNETGQESDDLYQELVQIQIE     310
Daphnia_pulex_SerRS2a                  SNV---------------QEERGIYRVHQFTKVEMFGVTSGEAA-ASDALLEEFVAIQKE     298
Capitella_teleta_SerRS2a               STV---------------DEERGIFRVHQFTKVEMFGVTANESGEESSQLLSQLIEIQKN     306
Anopheles_gambiae_SerRS2a              SAL---------------QEEKGIYRVHQFTKVEMFAVCQPG---QSATVLEEFRAIEVQ     299
Homo_sapiens_SerRS2                    NTG---------------QEPRGLYRVHHFTKVEMFGVTGPG-LEQSSQLLEEFLSLQME     377
Bos_taurus_SerRS2                      DTG---------------KEPWGLYRVHHFTKVEMFGVTGPG-LEQSSELLEEFLSLQME     372
Danio_rerio_SerRS2                     DTG---------------RETWGLYRVHHFTKIEMFGVSANETGEESSQLLDQFVTLQKE     404
Xenopus_tropicalus_SerRS2              DTG---------------RETWGLYRVHHFTKVEMFGVTANESGIESQEMLDEFLGLQKE     451
Caenorhabditis_elegans_SerRS2          SKS---------------ASEAKLYRVHEFSKVEMFVVSTPE---QSAAELDYLVEVQKG     316
Trichinella_spiralis_SerRS2            ATG---------------KLSKGLYRVHEFTKVEMFICTANETGLESNISHLEILEMQKL     297
Schistostoma_mansoni_SerRS2            PN-----------------QEPTLYRVHQFTKVEMFAITTPS-LPVSDAMFNQIIKLQIC     338
Intoshia_linea_SerRS2                  SPL---------------KSESGLYRVHDFSKVEMFGITENS-IEKSNLVFQEFLNIQNE     295
```

Supplementary Figure 2

Drosophila_melanogaster_SLIMP	FYKALDIPFRISYATAADLTPAESIRAVIEVYAPSL-------------QRYV**C**VG**R**ISN	391
Acanthaster_planci_SLIMP	LLSLLPLHGRIVEVPSARLLPSMYRKCAIEVWIPDA-------------GKFV**E**VA**S**VSN	457
Strongylocentrotus_purpuratus_SLIMP	LISTFDLPFRVVEVSSRNLLPCMHRQMAVELWIPSE-------------RNYI**E**VA**N**VCN	441
Triboleum_casteum_SLIMP	IYEPLNYHFRLVYAPAVDLKVGESLKVSIEMFSNYS-------------GTYV**E**VG**Y**LSL	380
Stegodyphus_mimosarum_SLIMP	CYQALGLPYRTVDVAAKNLIITESRRKQLELWSPSF-------------KKYV**P**VA**H**VSQ	412
Ramazzottius_varieronatus_SLIMP	FYLSLGLRFRLVSLPASKLGISEALRYELKVWSPLL-------------SEFV**P**CG**H**IAF	475
Pediculus_humanus_SLIMP	LYEQLGHHFRVVYVNASSLECWECLRASIQMYSCHE-------------QKYI**E**VG**N**ISI	401
Octopus_bimaculoides_SLIMP	FYSDLNLPVRLLLLPASQLHVSESKKAIIEMWAPSQ-------------QQYL**T**VA**S**VYL	438
Limulus_polyphemus_SLIMP	CYSLINLPFRIKKLASKNLGAAESLRIEFEVWAPAL-------------NDYA**L**CA**T**IIR	416
Lottia_gigantea_SLIMP	FLESFNLPIRQILVPVQDLSLSESLRSEFQMWSPSL-------------EDFV**N**MG**Y**LCM	427
Hyalella_azteca_SLIMP	AYQKLGLHFRIVRPEPRDLRVYERDALVVQMLSTSYPEGRNTVYIGENKMTYI**E**VG**R**ISI	630
Daphnia_pulex_SLIMP	QLQKLDVNFRIIAVPARQLESWEQYRSSVELFSSSS-------------KKFI**Q**VA**N**ISI	318
Capitella_teleta_SLIMP	FYEEIKIPIRINHVGSSSLRTSESQRIDFEVWSSAF-------------NRYF**K**IG**E**VSD	448
Anopheles_gambiae_SLIMP	FYDQLPVGYRIVQVPAPDLRPTESMRVDIEIFDGRN-------------QRHV**K**VA**E**LGY	371
Drosophila_melanogaster_SerRS2a	LFRRLGLNFRLLDMPPCELGAPAYQKYDIEAWMPGR-------------QMWG**E**IS**S**CSN	333
Acanthaster_planci_SRS2a	LFTELGLHFQVLDMPSHDLGAPAYRKYDIEAWMPAR-------------QSYG**E**IS**S**ASN	411
Strongylocentrotus_purpuratus_SerRS2a	LFSDLGLHFQILDMPAHDLGAPAYRKFDCEAWMPHR-------------KAYG**E**VS**S**TSN	430
Triboleum_casteum_SerRS2a	IFESLGLHFQVLDMPPHELGAQAYRKYDIEAWMPGR-------------GLYG**E**IS**S**CSN	343
Stegodyphus_mimosarum_SerRS2a	LYSELGLHFKILEMPPCELGLPAYHKIDMEAWIPTQ-------------KLYG**E**IS**S**ASN	298
Ramazzottius_varieronatus_SerRS2a	LVSTLGLHAQVLDMPKEELGLAASRKFDIEAWMPGS-------------QRYG**E**IS**S**ASN	238
Pediculus_humanus_SerRS2a	IFSSLGLDYQILDMPMHELGASAYRKHDIEAWFPGK-------------EIYG**E**IS**S**CSN	352
Octopus_bimaculoides_SerRS2	LYQSLGLHCRLLDMASQELGAPASRKFDYETWMPAK-------------GFWG**E**IS**S**ASN	378
Limulus_polyphemus_SerRS2a	LFQEIGLHFKLLDMPASDLGAPAYRKFDIEAWMPGR-------------GIYG**E**IS**S**ASN	364
Lottia_gigantea_SerRS2a	LFSQLGLHFRILEMSTECLGAPAYRKYDMETWMPGK-------------HFWG**E**IS**S**ASN	377
Hyalella_azteca_SerRS2a	LLQGLGLHGQVVLMPPEELGNPAYSKTDLEVWLPGR-------------RRYA**E**LT**S**ASN	357
Daphnia_pulex_SerRS2a	LFSSLGLHYQLLDMPLHELGAPAYRKFDIEAWMPGR-------------NMYG**E**IS**S**ASN	345
Capitella_teleta_SerRS2a	LFSSLDLHVRVLDMPTQELGAPATRKFDMEAWMPAK-------------KFWG**E**IS**S**ASN	353
Anopheles_gambiae_SerRS2a	LFDQLGLHFLLLDMPPCELGAPAYRKYDIEAWMPGR-------------AMYG**E**IS**S**CSD	346
Homo_sapiens_SerRS2	ILTELGLHFRVLDMPTQELGLPAYRKFDIEAWMPGR-------------GRFG**E**VT**S**ASN	424
Bos_taurus_SerRS2	ILTELGLHFRVLDMPTQELGLPAYRKFDIEAWMPGR-------------GRFG**E**VT**S**ASN	419
Danio_rerio_SerRS2	IFSSLQLHYRVLDMPTQELGPPAYRKFDIEAWMPGR-------------GSFG**E**IS**S**ASN	451
Xenopus_tropicalus_SerRS2	IFSELGLHFKVLEMPTQELGLPAYRKYDIEAWMPGR-------------GKYG**E**IS**S**TSN	498
Caenorhabditis_elegans_SerRS2	TFQALGVHCRQLEMPSEELGASAARKFDIEAWMPGR-------------KLYG**E**VS**S**ASN	363
Trichinella_spiralis_SerRS2	LFSELNLHFNVLEMPTEELGASAFRKFDIESWFPGR-------------DCYG**E**IS**S**ASN	344

Supplementary Figure 2

```
Drosophila_melanogaster_SLIMP            YGDFVSKRILFSTRRE-------------------KHYDFLHMV-----------GGPVL     421
Acanthaster_planci_SLIMP                 CTDYISRRVKMRYPAIPSQK-----------AAQSRQRNFPHTV-----------HCTAL     495
Strongylocentrotus_purpuratus_SLIMP      CTDFISRRLKIRYPTNPRES-----------AAQSRKRSFGHTI-----------HGTAL     479
Triboleum_casteum_SLIMP                  YGDYISKRLLFTHVTKP------------------PAFQYIILS-----------GATHL     411
Stegodyphus_mimosarum_SLIMP              RNNFLSKRLHITYGIQH------------------SVEGYCSIV-----------DGIVV     443
Ramazzottius_varieronatus_SLIMP          HDDFVSKRLRCHVDKP-------------------EQNKFLRFF-----------TGSLL     505
Pediculus_humanus_SLIMP                  SDKYICQRLLMSYQMADK----------------DDKSRFMKLV-----------SGTIV     434
Octopus_bimaculoides_SLIMP               TGTYIGERLMISSSSTQ------------------D--KVAHMV-----------HADLL     467
Limulus_polyphemus_SLIMP                 NGEYISKRLMMRHCSKG------------------NPNGFLHLI-----------HGTVV     447
Lottia_gigantea_SLIMP                    CDDFVSRRLMCLHDSKK------------------EGSMFVHMI-----------GGEVI     458
Hyalella_azteca_SLIMP                    IGDYISKRLLMMYVKDGARASSTSPRDGRAATGAVERLGFLQAV-----------YAQAM     679
Daphnia_pulex_SLIMP                      IGDYISRRLSIYG----------------------PNKEWPGFV-----------TSQAL     345
Capitella_teleta_SLIMP                   RSDFISRRLMITNSSNARGF---------------KEASPPFLI-----------HATLT     482
Anopheles_gambiae_SLIMP                  YTDFLSKRIAFIYHEG-------------------KVQRFPHVI-----------AGTAM     401
Drosophila_melanogaster_SerRS2a          CTDYQARRLGIRYRRSA-----------------DGQILHAHTI-----------NGTAT     365
Acanthaster_planci_SRS2a                 CTDYQSRRLRIKYRSK------------------EGDLRHAYTL-----------NGTAC     442
Strongylocentrotus_purpuratus_SerRS2a    CTDFQSRRLHIQYKPSP-----------------ESQAKYAHTL-----------NGTAC     462
Triboleum_casteum_SerRS2a                CLDYQARRLGIRTHD-----------------------GFVHTL-----------NGTAC     369
Stegodyphus_mimosarum_SerRS2a            CTDYQSRRLAIKYYNPL-----------------SNETKFCHTV-----------NGTAC     330
Ramazzottius_varieronatus_SerRS2a        CKDFQSSRLNITYQKKP--------------QETKDTLPFVHTV-----------NGTAC     273
Pediculus_humanus_SerRS2a                CIDYQSRRLNIKYRDE-------------------DKLFHVHTV-----------NGTAC     382
Octopus_bimaculoides_SerRS2              CTDYQSRRLGAKYK------------------NKADQLTYVHTI-----------NGTAC     409
Limulus_polyphemus_SerRS2a               CTDYQSRRLHIKYETQS--------------PDGRRVLKHVHTV-----------NGTAC     399
Lottia_gigantea_SerRS2a                  CTDYQSRRLGIKYT------------------DINGNYRHAHTV-----------NGTAC     408
Hyalella_azteca_SerRS2a                  CTSYQAARLGASYV------------------NKKGQRKLVHTV-----------NGTAV     388
Daphnia_pulex_SerRS2a                    CTDYQARRLNIQSI------------------DSSDTRRFVHTV-----------NGTAC     376
Capitella_teleta_SerRS2a                 CTDFQSRRLNIKYR------------------NSNGELGHCHTV-----------NGTGC     384
Anopheles_gambiae_SerRS2a                CTDYQARRLGIRVRTGQ-----------------EE--TFAHTV-----------NGTAC     376
Homo_sapiens_SerRS2                      CTDFQSRRLHIMFQ------------------TEAGELQFAHTV-----------NATAC     455
Bos_taurus_SerRS2                        CTDFQSRRLHIMFQ------------------TEAGELQFAHTV-----------NATGC     450
Danio_rerio_SerRS2                       CTDYQSRRLDIRYE------------------GEDGKLQYAHTV-----------NATAC     482
Xenopus_tropicalus_SerRS2                CTDYQSRRLNIMYQ------------------RQDGELRHAHTV-----------NGTAC     529
Caenorhabditis_elegans_SerRS2            CTDFQSRRLGIKYK------------------TADGTTKYAHTC-----------NGTAL     394
Trichinella_spiralis_SerRS2              CTDYQSRRLLTKYK------------------SSDGNLKFVHTVNKSIKLAKQNVNGTAC     386
```

Supplementary Figure 2

Drosophila_melanogaster_SLIMP	YTS**R**LIAALVEHGVRLE-----------DCKLLGSISQKPVHQQD-LQQ----------F	459
Acanthaster_planci_SLIMP	DTS**V**ILAALLPSVHQQL-PEV--LK---------PY----LPQAIPK-------------	526
Strongylocentrotus_purpuratus_SLIMP	KTS**V**LLSAMLDYGALDL-KEA--KS-----------------------------------	501
Triboleum_casteum_SLIMP	KSP--PNCSTSRLPQTA-PIY--NI--RHPVVLQAHVREPMTRITPLQN-----ATYAYL	459
Stegodyphus_mimosarum_SLIMP	NIP**V**LIACIMENFQTKN-YGF--EI--PP--VLKEIMDNLYA------------------	478
Ramazzottius_varieronatus_SLIMP	DLT**P**FVACCIDGSEGGI-FEH--IRRNPACVL-----------------------------	534
Pediculus_humanus_SLIMP	DVP**K**LLGCLIEEQEDED-KKF--PI--PDFLVI---------------------------	462
Octopus_bimaculoides_SLIMP	DVS**R**MLAFLIEHGKIGE-NEL--LQ--LP-----RLKLDAIPELIP--------------	503
Limulus_polyphemus_SLIMP	SIP**T**LIAAIIENCQTSD-CSF--VV--PK--VLQSYMLS---------------------	479
Lottia_gigantea_SLIMP	NIT**N**LLALMLEYDLP-------------------LISNK---------------------	478
Hyalella_azteca_SLIMP	DVT**R**VLAVAVENLQDAQ-GEA--NL--GK--LIDKLSS----------------------	710
Daphnia_pulex_SLIMP	SVP**K**LLYCYLKN--	357
Capitella_teleta_SLIMP	SIQ**P**LLAAILENSQSKD-GRI--CL--PG-----VY------------------------	508
Anopheles_gambiae_SLIMP	SSL**E**LIKLLLGNGITMQ-----------DLPMLNTLENE---------------------	429
Drosophila_melanogaster_SerRS2a	AIP**R**LLIALLESYQKED-G-I--EI--PA--VLRPFMDNQELITRNKRI-----PETKLV	412
Acanthaster_planci_SRS2a	AVP**R**VLLTLLECNQERD-GSV--SI--PK--ALQPYMNGKTVITKPPGG------FLHHT	489
Strongylocentrotus_purpuratus_SerRS2a	AVP**R**LIIAILENNQQRD-GSI--TI--PE--PLQPYMDGMREITRPASG------KLHFT	509
Triboleum_casteum_SerRS2a	AVP**R**LLIALVEMGQRKN-GTV--GL--PE--VLWPYMGGKQLIGKQKSV-----PDLRLI	417
Stegodyphus_mimosarum_SerRS2a	AIP**R**LLMTIFENFQNLD-GSI--TV--PA--PLQKYMGKDVI-KEKFS-----E-KI--L	374
Ramazzottius_varieronatus_SerRS2a	AVP**R**MIIAILEQNQLSN-GII--TI--PE--VLRRYFDNAVTLSKPTGA----T-GLYWR	321
Pediculus_humanus_SerRS2a	AIP**R**MLMALFETHQQQN-GNI--LL--PK--PLRPFLNGKEVIQVQNSI-----PKLISR	430
Octopus_bimaculoides_SerRS2	AVP**R**LVMAIIENNQLPN-GSV--LL--PQ--VLHPYFQGSLLTVEPK-------SLNHWK	455
Limulus_polyphemus_SerRS2a	AIP**R**MLIAILENFQTKD-GDI--RI--PD--PLIPHMRGLAVIKNRRTK----D-KIYWT	447
Lottia_gigantea_SerRS2a	AIP**R**MIMAILEQFQTKD-WNV--NI--PE--VLQPYMNNKTQLTGPQNK----H-PTKWI	456
Hyalella_azteca_SerRS2a	AVP**R**CIIALCETHQEAT-GRRALQL--PA--FLGLAPAHPQQFQPNETSVDTGHEHNAFL	443
Daphnia_pulex_SerRS2a	AVP**R**TLIALLETHQQSN-GEV--II--PA--VLQPYLNGMQRLRNPTQP----F-KMKWI	424
Capitella_teleta_SerRS2a	AVP**R**VLMSLIENNQQSD-GTV--VI--PT--ALRSFMAGQQLISASEDV----LPNTHWI	433
Anopheles_gambiae_SerRS2a	AIP**R**MLIALLENFQNED-YTI--TV--PE--VLRRYMGGKQLLRRRKVL-----PELKLT	424
Homo_sapiens_SerRS2	AVP**R**LLIALLESNQQKD-GSV--LV--PP--ALQSYLGTDRITAPTHV-------PLQYI	501
Bos_taurus_SerRS2	AVP**R**LLIALLESYQQKD-GSV--LV--PP--ALQPYLGTDRITTPTHV-------PLQYI	496
Danio_rerio_SerRS2	AIP**R**TIIAILETYQTKK-GTV--QV--PE--VLQQYIGMEVIEKPKYT-------PIKYI	528
Xenopus_tropicalus_SerRS2	AVP**R**LLIAILESNQRKD-GAV--LI--PE--VLQPYMGTDVIEKPRYI-------PARYI	575
Caenorhabditis_elegans_SerRS2	AST**R**ALISVLETFQNDKKGLG--EL--PE--PLRKRVKQRGS-------------PLRFQ	435
Trichinella_spiralis_SerRS2	ALS**R**TLIAMLEQFQCKN-RQV--NI--PK--FLLDYVNVEMKRQYAERK------PLSLL	433

Supplementary Figure 3

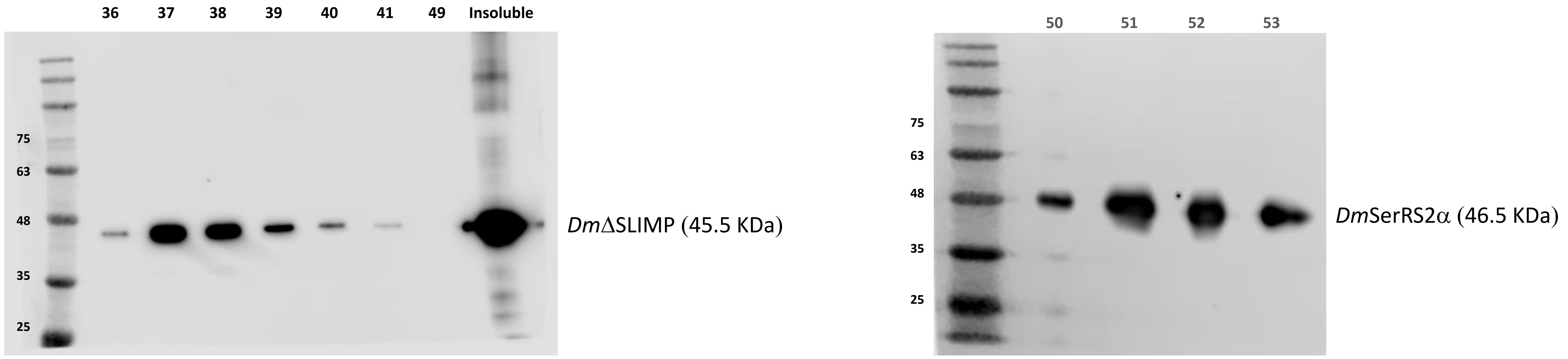

Western blot analyses of purified 6His-tagged *Dm*ΔSLIMP and *Dm*SerRS2α using an Anti-His antibody.
These proteins were used to attempt the reconstitution of the *Dm*SerRS2α/ *Dm*ΔSLIMP complex.
